# Co-targeting of the thymic stromal lymphopoietin receptor to decrease immunotherapeutic resistance in *CRLF2*-rearranged Ph-like and Down syndrome acute lymphoblastic leukemia

**DOI:** 10.1038/s41375-024-02493-3

**Published:** 2024-12-16

**Authors:** Tommaso Balestra, Lisa M Niswander, Asen Bagashev, Joseph P Loftus, Savannah L Ross, Robert K Chen, Samantha M McClellan, Jacob J Junco, Diego A Bárcenas López, Karen R. Rabin, Terry J Fry, Sarah K Tasian

**Affiliations:** 1 Division of Oncology and Center for Childhood Cancer Research, Children’s Hospital of Philadelphia, Philadelphia, PA USA; 2 Department of Pediatrics, University of Pennsylvania, School of Medicine, Philadelphia, PA USA; 3 Division of Hematology/Oncology/Bone Marrow Transplant and Center for Cancer and Blood Disorders, Children’s Hospital of Colorado, Aurora, CO USA; 4 Texas Children’s Hospital Cancer Center and Division of Pediatric Hematology/Oncology, Houston, TX USA; 5 Department of Pediatrics, Baylor College of Medicine, Houston, TX USA; 6 University of Colorado Anschutz Medical Campus and Gates Institute, Aurora, CO USA; 7 Abramson Cancer Center, University of Pennsylvania, School of Medicine, Philadelphia, PA USA; 8 Prinses Máxima Center for Pediatric Oncology, Utrecht, The Netherlands

**Keywords:** Translational research, Acute lymphocytic leukaemia, Paediatrics, Immunotherapy

## Abstract

*CRLF2* rearrangements occur in >50% of Ph-like and Down syndrome (DS)-associated B-acute lymphoblastic leukemia (ALL) and induce constitutive kinase signaling targetable by the JAK1/2 inhibitor ruxolitinib under current clinical investigation. While chimeric antigen receptor T cell (CART) immunotherapies have achieved remarkable remission rates in children with relapsed/refractory B-ALL, ~50% of CD19CART-treated patients relapse again, many with CD19 antigen loss. We previously reported preclinical activity of thymic stromal lymphopoietin receptor-targeted cellular immunotherapy (TSLPRCART) against *CRLF2*-overexpressing ALL as an alternative approach. In this study, we posited that combinatorial TSLPRCART and ruxolitinib would have superior activity and first validated potent TSLPRCART-induced inhibition of leukemia proliferation in vitro in *CRLF2-*rearranged ALL cell lines and in vivo in Ph-like and DS-ALL patient-derived xenograft (PDX) models. However, simultaneous TSLPRCART/ruxolitinib or CD19CART/ruxolitinib treatment during initial CART expansion diminished T cell proliferation, blunted cytokine production, and/or facilitated leukemia relapse, which was abrogated by time-sequenced/delayed ruxolitinib co-exposure. Importantly, ruxolitinib co-administration prevented fatal TSLPRCART cytokine-associated toxicity in ALL PDX mice. Upon ruxolitinib withdrawal, TSLPRCART functionality recovered in vivo with clearance of subsequent ALL rechallenge. These translational studies demonstrate an effective two-pronged therapeutic strategy that mitigates acute CART-induced hyperinflammation and provides potential anti-leukemia ‘maintenance’ relapse prevention for *CRLF2*-rearranged Ph-like and DS-ALL.

## Introduction

Ph-like ALL is a high-risk subset of B-acute lymphoblastic leukemia (ALL) defined by an activated kinase gene expression profile similar to that of *BCR::ABL1*-rearranged (Ph+) ALL and driven by a diverse range of other genetic alterations that activate cytokine receptor signaling pathways [1–3]. Children, adolescents/young adults (AYAs), and older adults with Ph-like ALL have >60% relapse risk and experience significant leukemia-associated mortality with current best-available conventional chemotherapy [2, 4, 5]. Approximately 50% of Ph-like ALL cases harbor rearrangements in *CRLF2* and have frequent concomitant *JAK2* or *JAK1* point mutations [6, 7]. *CRLF2* rearrangements and activating JAK mutations also occur in 50–60% of Down-syndrome-associated ALL (DS-ALL) cases [6–9].

The CRLF2 protein heterodimerizes with the IL7R alpha chain (CD127) to form the heterodimeric thymic stromal lymphopoietin receptor (TSLPR) that is aberrantly overexpressed at high levels on the surface of *CRLF2*-rearranged (*CRLF2*-R) B-ALL cells and is easily detectable by flow cytometric immunophenotyping [10]. Our group and others previously identified constitutive JAK/STAT, PI3K/mTOR, and BCR-like/SFK signaling in primary Ph-like ALL cells and associated patient-derived xenograft (PDX) models, providing rationale for development of tyrosine kinase inhibitor (TKI)-based therapies that may improve clinical outcomes for patients with these chemoresistant leukemias [10–14]. However, during the past decade, we have also observed heterogeneous in vitro and in vivo responses of preclinical *CRLF2*-R ALL models to JAK inhibitor (JAKi) treatment with ruxolitinib monotherapy, indicating that this subtype of Ph-like ALL may not be solely JAK oncogene-addicted [13, 15, 16]. While various JAKi and other TKIs have been empirically tested in the laboratory, the mechanisms of potential resistance to these drugs in Ph-like ALL remain poorly understood [10, 17, 18]. Subsequent efforts have thus focused upon combination therapy approaches [13, 19, 20]. However, whether addition of ruxolitinib to chemotherapy under evaluation in current clinical trials (NCT02723994, NCT03571321) will decrease relapse risk for patients with *CRLF2*-rearranged/JAK pathway mutant Ph-like ALL and what resistance mechanisms may develop in JAKi-treated patients remain major knowledge gaps [3]. Importantly, this question has remained unasked and thereby unanswered in patients with DS-ALL, who have been excluded from such trials to date [21].

CD19-directed chimeric antigen receptor T cell (CD19CART) immunotherapy induced initial remissions in 80–90% of patients with multiply-relapsed/refractory B-ALL treated on institutional phase 1 trials [22–24], leading to FDA approval of the CD19CART tisagenlecleucel for children and AYAs in second or greater relapse of B-ALL. Current ‘real world’ usage, including in patients with adverse-risk Ph-like genetics and patients with DS-ALL, has validated the effectiveness of this therapy [25–27]. Although durable remissions with CD19CART monotherapy are possible, 50% of patients achieving CD19CART-induced ALL remission relapse again within 1 year [25]. While earlier (usually CD19+) ALL relapses are typically associated with short functional CD19CART persistence, later relapses are highly associated with target antigen loss and immunotherapeutic resistance [28, 29]. CAR T cell immunotherapies also mediate potentially life-threatening hyperinflammation, including cytokine release syndrome (CRS) [30, 31]. Targeting of excessive IL-6 production with anti-IL-6 receptor monoclonal antibody therapies has been effective at treating high-grade CRS driven by CAR T cell activation and proliferation without compromising desired anti-leukemia activity and longer-term CAR T cell persistence [32, 33]. However, prophylactic administration of IL-6-targeted agents has shown limited success to date and may increase risk of neurotoxicity [34]. Successful approaches to overcome both immunotherapeutic resistance to CAR T cells and their associated toxicities thus has potential to improve clinical outcomes of treated patients.

We previously developed TSLPR-targeted CAR T cell immunotherapy (TSLPRCART) and demonstrated robust and sustained activity in vivo in CRLF2-overexpressing (CRLF2+) ALL xenograft models [35]. Given the mutational profiles and constitutive kinase signaling in both Ph-like ALL and DS-ALL, we hypothesized in the current study that co-targeting of (1) the extracellular highly-expressed cell surface TSLPR with CAR T cell immunotherapy and (2) intracellular JAK/STAT signaling with ruxolitinib could have synergistic anti-leukemia activity in CRLF2+ Ph-like and DS-ALL. We further postulated that ruxolitinib might also directly fine-tune deleterious inflammatory effects of CAR T cell hyperactivation given importance of JAK signaling in the cytokine response effectiveness in the treatment of patients with graft-versus-host disease [36] or hemophagocytic lymphohistiocytosis [37]. Herein, we report potent in vitro and in vivo activity of TSLPRCART immunotherapy and JAK inhibition in preclinical models of *CRLF2*-rearranged Ph-like ALL and DS-ALL with reversible ruxolitinib-mediated suppression of TSLPRCART-induced CRS-like inflammatory responses [38, 39]. These studies highlight the importance of optimal sequencing of TSLPRCART immunotherapy and ruxolitinib in the context of planned phase 1 clinical trials for children and AYAs with relapsed/refractory CRLF2+ leukemias.

## Methods

### Chimeric antigen receptor T cell production

Second-generation TSLPRCART and CD19CART (both with 4-1BB/CD3ζ costimulatory domains) were designed as previously described and as detailed in Supplementary Methods [35, 40]. Human T cells from four healthy donors obtained from the University of Colorado or University of Pennsylvania human immunology core facilities were utilized in these studies to ensure robustness and reproducibility. Experimental dosing of TSLPRCART and CD19CART was based upon total T cell numbers given consistent CAR transduction efficiency of 50–70% for all products utilized in these studies.

### Human ALL cell lines and patient-derived xenograft models

*CRLF2-*rearranged MUTZ5 (TSLPR+/CD19+) and *CRLF2* wild-type NALM-6 (TSLPR-/CD19+) human ALL cell lines were purchased from the DSMZ cell line biorepository (Braunschweig, Germany). Cell lines were cultured for no longer than 2 months in RPMI medium containing 10% or 20% heat-inactivated fetal bovine serum, 2 mM L-glutamine, and 100 U/mL penicillin/streptomycin and regularly confirmed to be *Mycoplasma*-free. Cell line authentication was performed by short tandem repeat profiling and fluorescence in situ hybridization analysis of known chromosomal translocations. *CRLF2*-rearranged Ph-like ALL and DS-ALL PDX models in Table 1 were created using primary ALL cells as previously described and in Supplementary Methods [13, 19, 41]. Luciferase-transduced ALL cells were created as described for in vivo xenograft studies utilizing bioluminescent imaging assessment [35, 40].

**Table 1:** Genetic characteristics of *CRLF2*-rearranged ALL cell line and patient-derived xenograft models utilized in these studies.

Xenograft model	COG USI	Disease status	*CRLF2* rearrangement	Other genetic alterations
MUTZ5	n/a	newly-diagnosed Ph-like ALL	*IGH::CRLF2*	*JAK2* R683G mutation
JH331	PAMDKS	newly-diagnosed Ph-like	*IGH::CRLF2*	*CDKN2A*, *IKZF1*, *PAX5* deletions
ALL121	none	relapsed Ph-like ALL	*IGH::CRLF2*	*CDKN2A/B* deletion, *JAK2* R683G mutation
DS-ALL47	PAUVIE	newly-diagnosed DS-ALL	*P2RY8::CRLF2*	*JAK2* L730F, K926R, I951K mutations
DS-ALL515	PAWBHJ	relapsed DS-ALL	*P2RY8::CRLF2*	*BTG1*, *CDKN2A/B*, *IKZF1*, *PAX5*, *PMS2*, *TCRG* deletions
TCHK150	none	relapsed DS-ALL	*IGH::CRLF2*	*JAK2* R867Q mutation, *SCMH1::HMHB1*, *NR3C1::NWD2*, *C19orf60::ZCCHC7* fusions

*COG USI* Children’s Oncology Group unique specific identifier, *DS-ALL* Down syndrome-associated acute lymphoblastic leukemia, *n/a* not applicable, *Ph-like ALL* Philadelphia chromosome-like acute lymphoblastic leukemia.

### In vitro evaluation of TSLPRCART activity

CD3/CD28 bead-stimulated normal donor T cells were incubated in vitro without and with ruxolitinib (LC Laboratories) at concentrations noted in the figure legends. Quantification of T cell expansion and CD4+/CD8+ subsets by flow cytometry analysis, viability by a luciferase reporter viability assay (Promega; Madison, Wisconsin), and IL-2 and IFN-γ cytokine production by ELISA were performed as previously described [40, 42]. Each control and experimental condition was plated in technical triplicate. Co-culture experiments with TSLPRCART or CD19CART and luciferase+ ALL cell lines were performed as described and as detailed in Supplementary Methods [35, 40, 42].

### In vivo evaluation of TSLPRCART and ruxolitinib anti-leukemia activity in xenograft models

Human ALL cell line xenograft and PDX model studies in NOD-scid IL2Rγ^null^ (NSG) mice were conducted via an Institutional Animal Care and Use Committee-approved research protocol at the Children’s Hospital of Philadelphia. Cohorts of ALL-engrafted animals (*n* = 5 or 10) were randomized to treatment with vehicle (saline), mock-transduced T cells, TSLPRCART, CD19CART, and/or ruxolitinib at the doses, routes, and timing noted in the figure legends and Supplementary Methods and as previously described [13, 19, 41].

### Flow cytometry analyses

Flow cytometric quantification and characterization of human ALL and T cells from in vitro and in vivo studies were performed using BD FACSVerse or Beckman Coulter Cytoflex flow cytometers and analyzed with Cytobank or FlowJo software as described [40]. TSLPR cell surface molecules/cell enumeration was performing using TSLPR-PE antibodies (Invitrogen #12-5499-42; Waltham, Massachusetts) and QuantiBrite-PE beads (BD Biosciences #C36995; La Jolla, California) as described [10, 40]. Other flow cytometry antibodies and additional experimental details are listed in Supplementary Methods.

### Statistical analyses

Statistical analyses, data normality assessment, and data display were performed using Prism software (GraphPad; Carlsbad, California). Two-tailed unpaired Student *t* tests and one-way or two-way analysis of variance (ANOVA) with Dunnett or Tukey post-tests for multiple comparisons were performed as indicated in the figure legends. Data are reported as mean values ± standard error of the mean (SEM). Statistical significance is indicated as ns = not significant, *p < 0.05, **p < 0.01, ***p < 0.001, and ****p < 0.0001 in the relevant figure legends.

## Results

### TSLPRCART and ruxolitinib co-administration protects against CAR T cell-induced toxicity, but blunts anti-leukemia efficacy

**Fig. 1:**
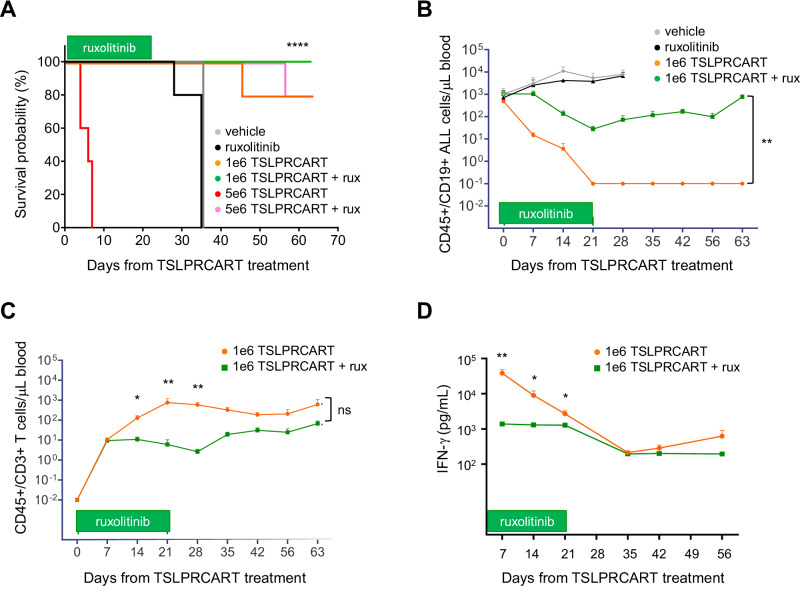
Simultaneous JAK inhibition ameliorates in vivo CAR T cell-induced mortality and suppresses TSLPRCART activity in ruxolitinib-insensitive *CRLF2-*rearranged Ph-like ALL. **A** Kaplan-Meier survival analysis of *IGH::CRLF2* Ph-like ALL patient-derived xenograft (PDX) mice (JH331 model). Cohorts of 5 mice were randomized and treated intravenously (IV) with 5e6 TSLPRCART or vehicle (saline) control with or without simultaneous exposure to ruxolitinib 2 g/kg rodent chow administered continuously (*ad libitum*) days 0 through 21 (horizontal green bar). **B** Human CD45+/CD19+B-ALL cells and (**C**) human CD45+/CD3+CAR T cells were quantified weekly via flow cytometric analysis of sampled peripheral blood from JH331 mice treated with vehicle, ruxolitinib monotherapy, or lower-dose 1e6 TSLPRCART with or without simultaneous ruxolitinib treatment from days 0 to day 21 (green bar). Lower TSLPRCART numbers are initially detected at early ruxolitinib co-administration timepoints, then normalize in subsequent weeks after ruxolitinib withdrawal. **D** IFN-γ levels in plasma from JH331 mice treated with 1e6 TSLPRCART in (**B**) and (**C**) are lower in mice treated with simultaneous ruxolitinib. Depicted data represent mean ± standard error of the mean (SEM). Statistical analyses were performed with Kaplan-Meier survival analysis with log-rank (Mantel-Cox) for comparison, 2-way ANOVA with Šidák correction, or unpaired *t*-tests at relevant time points. ns not significant, *p < 0.05, **p < 0.01, ****p < 0.0001.

Given the anticipated direct impact of JAK inhibition upon CAR T cells, we first asked whether ruxolitinib would affect in vivo TSLPRCART fitness and anti-leukemia activity utilizing a known surprisingly ruxolitinib-insensitive *IGH::CRLF2* childhood Ph-like ALL PDX model with high TSLPR surface expression (JH331 from Children’s Oncology Group [COG] unique specimen identifier PAMDKS) [15, 19]. Unexpectedly, higher-dose TSLPRCART (5e6 cells; previously with demonstrated efficacy in *CRLF2*-overexpressing ALL cell line xenograft mice [35]) treatment of JH331 PDX mice resulted in universal fatality within 1 week (Fig. 1A, orange) thought to be potentially attributable to a cytokine-associated hyperinflammatory process. Although 21 days of ruxolitinib monotherapy had no appreciable effect upon animal survival compared to vehicle treatment (Fig. 1A, black versus gray), co-administration of ruxolitinib with 5e6 TSLPRCART strikingly resulted in long-term survival (Fig. 1A, green), suggesting mitigation of life-threatening inflammation and benefit of combinatorial therapy. Given the toxicity of higher-dose TSLPRCART in this PDX model, we then utilized lower-dose TSLPRCART (1e6 cells) in subsequent experiments to delineate better the potential additive or synergistic effects of simultaneous ruxolitinib administration upon TSLPRCART efficacy, a scenario in which greater T cell proliferative capacity is required. Consistent with our prior study [35], lower-dose TSLPRCART monotherapy completely eradicated detectable human ALL in murine peripheral blood (Fig. 1B, orange) with notable peripheral blood T cell expansion and persistence (Fig. 1C, orange) and peak plasma IFN-γ levels at one week post-treatment (Fig. 1D, orange), as well as long-term animal survival. Conversely, concurrent co-administration of ruxolitinib diminished TSLPRCART-mediated inhibition of in vivo CRLF2+ ALL proliferation (Fig. 1B, green), T cell expansion in peripheral blood (Fig. 1C, green), and IFN-γ production (Fig. 1D, green). Interestingly, subsequent ruxolitinib withdrawal at day 21 allowed partial spontaneous recovery of TSLPRCART proliferation in peripheral blood (Fig. 1C). These results indicate that JAK1/2 inhibition dampens desired TSLPRCART anti-leukemia activity in vivo, but also prevents deleterious high-dose CART-induced toxicity and mortality.

### Ruxolitinib inhibits T cell expansion, activation, and cytokine production

**Fig. 2:**
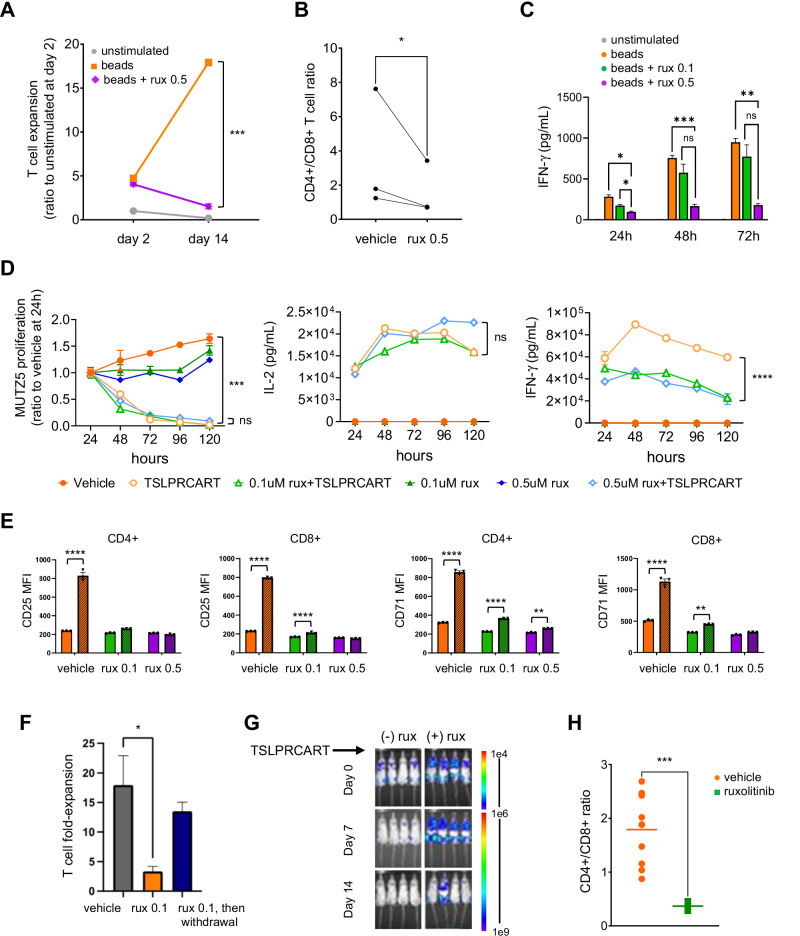
Ruxolitinib co-administration inhibits T cell expansion and cytokine production and preferentially affects CD4 + T cells. **A** Normal T cells from healthy donors (*n* = 3 donors) were cultured with CD3/CD28 beads with or without ruxolitinib (0.5 µM) in vitro for 2 weeks, with unstimulated T cells cultured without CD3/CD28 beads as a control. T cell expansion was assessed via Cell Titer Glo viability assays. Depicted data represent the mean of 3 independent T cell donors plated in technical triplicates ±SEM. **B** The ratio of CD4/CD8+ T cells was measured by flow cytometric immunophenotyping of normal healthy donor T cells exposed to 0.5 μM ruxolitinib for 72 h. **C** IFN-γ in culture supernatant of CD3/CD28 bead activated T cells treated with or without ruxolitinib at the indicated concentrations was quantified by ELISA at the indicated timepoints. Depicted data represent the mean of 3 independent T cell donors plated in technical triplicates ±SEM. **D** Luciferase-transduced MUTZ5 (a TSLPR+ human *CRLF2*-rearranged ALL cell line) cells were co-incubated in vitro with TSLPRCART at 1:15 effector-to-target (E:T) ratio and either vehicle or ruxolitinib at 0.1 and 0.5 μM concentrations. ALL cytotoxicity via luciferase reporter assays (left) and IL-2 (middle) and IFN-γ (right) production via ELISA were measured at the indicated time points. Depicted data represent the mean of technical triplicates ± SEM. **E** TSLPRCART were incubated in the absence (solid bars) or presence (striped bars) of MUTZ5 B-ALL cells in 1:1 E:T ratio for 24 h with or without ruxolitinib at the indicated concentrations. Induction of CD25 (left two panels) or CD71 (right two panels) surface expression on CD4+ and CD8+ T cells was evaluated by flow cytometry analysis. Quantification of median fluorescent intensity (MFI) for CD4+ and CD8+ T cell subsets with technical triplicates for each condition is displayed ±SEM. Ruxolitinib blunted upregulation of cell surface T cell activation markers CD25 and CD71 on both CD4+ and CD8+ TSLPRCART when co-incubated with MUTZ5 cells. **F** TSLPRCART were co-incubated 1:1 with MUTZ5 cells with or without ruxolitinib at the indicated concentrations. Every 3–4 days, TSLPRCART were sampled for enumeration by quantitative flow cytometry analysis to determine expansion from prior plating, and the remaining cells were re-stimulated 1:1 with MUTZ5 and fresh ruxolitinib-containing media. On day 7, a subset of ruxolitinib-exposed TSLPRCART were replated in the absence of ruxolitinib (withdrawal condition) for 4 days, and this ruxolitinib-induced decrease in in vitro TSLPRCART expansion was observed to be reversible upon drug removal. Depicted data represent the mean of technical triplicates ±SEM. **G** Ruxolitinib co-administration also inhibited anti-leukemia activity of TSLPRCART in vivo in a bioluminescent *IGH::CRLF2*/*JAK2*^R683^-mutant Ph-like ALL PDX model (ALL121). Luciferase-expressing ALL121 cells were injected (1e6) IV into NSG mice. Once engraftment was confirmed by bioluminescent imaging, cohorts of 4–8 mice were treated with 1e6 TSLPRCART IV and simultaneously exposed to ruxolitinib-infused chow (rux) or control rodent chow. **H** At day 14 of the experiment in (**G**), human CD4+ and CD8+ T cells in murine peripheral blood were quantified by flow cytometry and demonstrate significantly decreased CD4:CD8 ratio. Depicted data represent mean ±SEM. After data normality assessment, statistical analyses were performed for (**A**) and (**C**) with two-way ANOVA with Tukey post-test for multiple comparisons, for (**B**) with a paired *t*-test, for (**D**) with one-way ANOVA and Dunnett post-test for multiple comparisons using the TSLPRCART condition as the comparator, for (**E**) and (**F**) with one-way ANOVA and Tukey post-test for multiple comparisons, and for (**H**) with an unpaired *t*-test. ns not significant, *p < 0.05, **p < 0.01, ***p < 0.001, ****p < 0.0001.

To investigate the mechanism(s) of JAKi-mediated T cell inhibition, we treated CD3/CD28 bead-stimulated normal donor T cells with ruxolitinib in vitro. Concordant with other reports [43], we confirmed by Western blotting that ruxolitinib exposure abrogated phosphorylation of STAT5 and ERK1/2 in protein lysates from control- and ruxolitinib-exposed T cells (Supplementary Fig. 1A) and significantly impaired in vitro T cell expansion (Fig. 2A). This suppression was notably more pronounced in CD4+ T cells, resulting in an altered CD4:CD8 T cell ratio (Supplementary Fig. 1B–D; Fig. 2B). In addition, ruxolitinib reduced IFN-γ production in a dose-dependent manner (Fig. 2C). We next examined the effects of ruxolitinib co-exposure upon TSLPRCART activity against CD19+ *CRLF2*-rearranged Ph-like B-ALL MUTZ5 cells in vitro. Interestingly, ruxolitinib did not impair TSLPRCART-induced cytotoxicity in viability assays, but significantly diminished IFN-γ production (Fig. 2D). These observations were recapitulated in additional in vitro non-*CRLF2*-rearranged NALM-6 experiments with CD19CART and ruxolitinib co-treatment (Supplementary Fig. 1E, F), supporting that ruxolitinib-mediated T cell effects are not restricted to TSLPRCART. Exposure to ruxolitinib also blunted expression of T cell early activation markers CD25 and CD71 in both CD4+ and CD8+ subsets of TSLPRCART in MUTZ5 co-culture assays (Fig. 2E). Moreover, short-term treatment with ruxolitinib negatively affected TSLPRCART polyfunctionality (defined as a single cell secreting at least 2 of 32 measured functionally relevant molecules) [44] (Supplementary Fig. 2).

In co-culture experiments, repetitive in vitro stimulation with MUTZ5 in the presence of ruxolitinib suppressed TSLPRCART expansion. Importantly, TSLPRCART proliferative capacity recovered fully over time after ruxolitinib withdrawal, suggesting reversibility of T cell suppression (Fig. 2F). To validate these in vitro observations, we treated another *IGH::CRLF2*/*JAK2*^R683G^-mutant Ph-like ALL PDX model (luciferase+ ALL121 [19]) with simultaneous ruxolitinib and TSLPRCART. As anticipated, JAKi co-treatment suppressed in vivo CAR T cell activity with delayed leukemia clearance compared to TSLPRCART and vehicle treatment (Fig. 2G) and led to preferential decrement in CD4+ T cell numbers (Fig. 2H).

### JAKi-mediated inhibition of in vivo TSLPRCART proliferation and anti-leukemia activity is reversible and mitigated by delayed co-administration of ruxolitinib

**Fig. 3:**
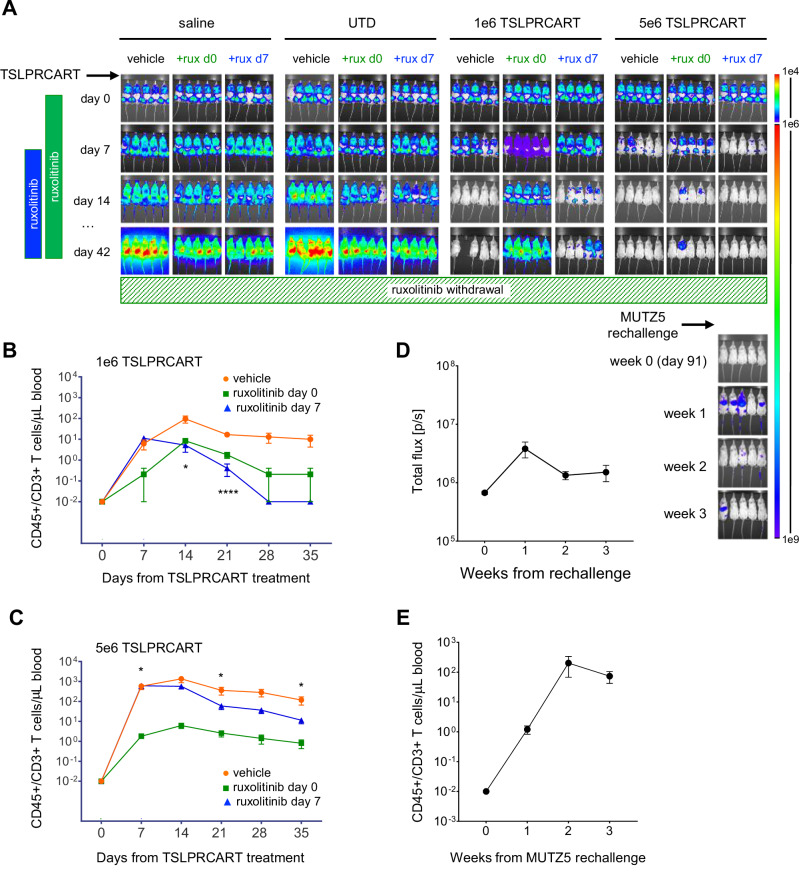
Ruxolitinib-induced inhibition of TSLPRCART is improved with delayed co-administration and is reversible. **A** Luciferase-transduced MUTZ5 cells were injected IV into NSG mice. Once engraftment was documented by bioluminescent imaging (BLI), cohorts of 5 mice were randomized to IV treatment on day 0 with saline, 1e6 untransduced T cells (UTD), or lower-dose (1e6) or higher-dose (5e6) TSLPRCART. Ruxolitinib chow (rux) *ad libitum* was administered simultaneously at day 0 (green) or day 7 after T cell treatments (blue) and continued to day 42. Leukemia burden was measured weekly by BLI. Human CD3+/CD45+ T cells were quantified weekly by flow cytometry analysis of peripheral blood of mice treated with **B** lower-dose or **C** higher-dose TSLPRCART. Statistical analysis was performed by two-way ANOVA with Tukey post-test for multiple comparisons with differences indicated at relevant timepoints. **D** Ruxolitinib was then removed at day 42 (striped green bar) for relevant cohorts, and mice continued to be followed by BLI to monitor potential re-emergence of leukemia. After 49 additional days without ruxolitinib exposure (day 91), mice were injected IV with 1e7 luciferase-expressing TSLPR + MUTZ5 cells to simulate relapse (week 0 antigen rechallenge) and followed by BLI. **D** Summary BLI radiance data following MUTZ5 rechallenge are displayed graphically for the 5e6 TSLPRCART/original ruxolitinib day 7 cohort shown in the lower right aspect of (**A**) with **(E)** enumeration of human CD3+/CD45+ T cells in murine peripheral blood at these same time points by quantitative flow cytometry. TSLPRCART with prior day 7 delayed-ruxolitinib exposure expanded robustly following MUTZ5 rechallenge, and low or undetectable leukemia burden was maintained. Statistical analyses were performed for (**B**) and (**C**) with one-way ANOVA and Tukey post-test for multiple comparisons. *p < 0.05, ****p < 0.0001.

Given the observed suppressive effects of JAKi upon T cell proliferation, we hypothesized that delaying administration of ruxolitinib after achievement of initial CAR T cell expansion could minimize its deleterious effects upon T cell functionality and potentially also serve as an anti-leukemia maintenance therapy strategy. As we have previously shown in vitro [10], *IGH*::*CRLF2/JAK2*^R683^-mutant Ph-like ALL MUTZ5 cells were only partially sensitive to ruxolitinib monotherapy in vivo in a newly-created luciferase+ cell line xenograft model. In these first studies, ruxolitinib initiation at day 7 (Fig. 3A–C, blue) after TSLPRCART treatment of MUTZ5 xenograft mice resulted in greater TSLPRCART expansion and IFN-γ production in peripheral blood compared to simultaneous (day 0) co-treatment with concomitantly improved ALL clearance (Fig. 3A–C, green; Supplementary Fig. 3). Time-sequenced ruxolitinib initiation on day 7 in combination after lower-dose 1e6 TSLPRCART administration on day 0 improved ALL clearance compared to mice receiving concurrent (day 0) ruxolitinib treatment, but nonetheless resulted in a suboptimal treatment response compared to TSLPRCART alone. At the higher 5e6 TSLPRCART dose, however, sequenced ruxolitinib therapy initiated on day 7 was completely curative, while day 0 simultaneous co-therapy was not. To test if ruxolitinib-exposed TSLPRCART could retain or recover in vivo persistence and functionality capabilities, ruxolitinib was then stopped following five or 6 weeks of co-administration (Fig. 3A, striped green bar). Six weeks following ruxolitinib discontinuation, mice in previously TSLPRCART-induced ALL remission were rechallenged with MUTZ5 (Fig. 3A far right lower panel), which resulted in re-expansion of TSLPRCART in peripheral blood and leukemia re-control in most mice (Fig. 3D, E). These results demonstrate that the observed suppressive effects of ruxolitinib upon desired anti-leukemia TSLPRCART activity may be abrogated with delayed ruxolitinib co-administration and, notably, appear fully reversible upon ruxolitinib withdrawal.

### Sequenced ruxolitinib co-therapy after peak TSLPRCART expansion in vivo enhances long term remission and survival

**Fig. 4:**
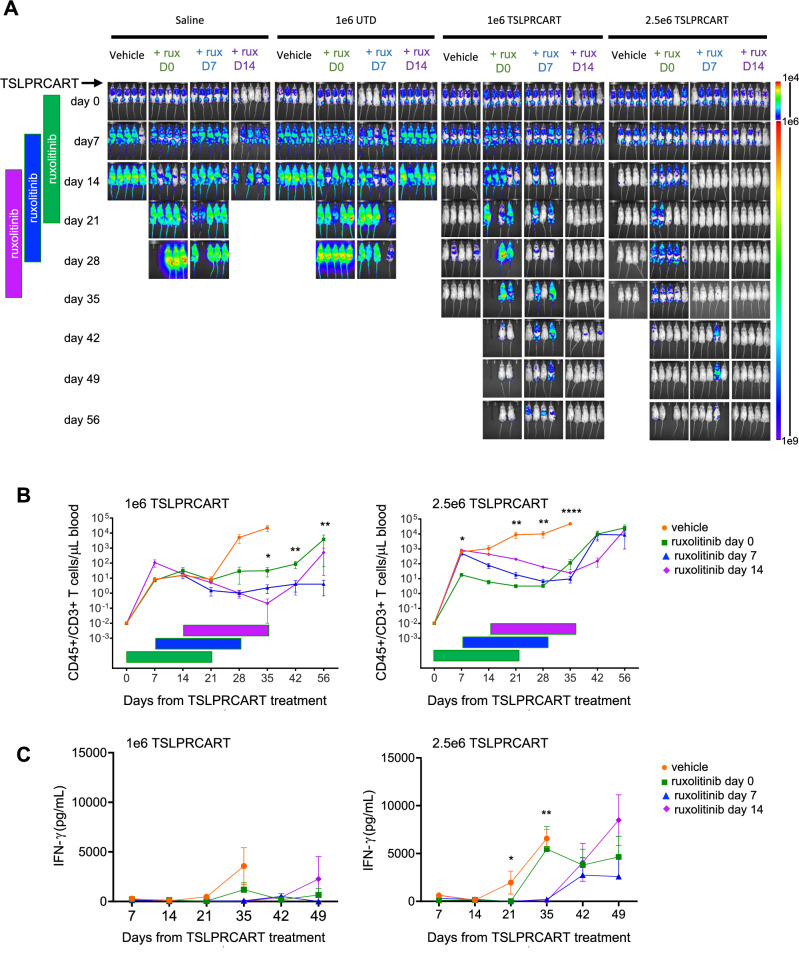
Delayed JAK inhibitor co-treatment improves in vivo TSLPRCART activity against a ruxolitinib-sensitive *CRLF2*-rearranged Ph-like ALL PDX model. **A** Luciferase-transduced *IGH::CRLF2*/*JAK2*^R683G^-mutant ALL121 PDX model cells (1e6) were injected IV in NSG mice. Once engraftment was documented by BLI, cohorts of 5 mice were randomized to IV treatment with saline, 1e6 untransduced T cells (UTD), or lower-dose (1e6) or higher-dose (2.5e6) TSLPRCART. Ruxolitinib (rux) chow *ad libitum* was administered simultaneously at day 0 (green), day 7 (blue), or day 14 (purple) after T cell treatments and continued for 21 days in each cohort until days 21, 28, or 35, respectively. Leukemia burden was measured weekly by BLI. **B** Human CD3+/CD45+ T cells were quantified weekly by flow cytometry analysis of peripheral blood of mice treated with lower-dose (left panel) or higher-dose (right panel) TSLPRCART. **C** IFN-γ was measured by ELISA in plasma prepared from weekly peripheral venous blood from mice treated with lower-dose (left panel) or higher-dose (right panel) TSLPRCART. Time-sequenced ruxolitinib co-administration at day 14 (at initial peak of detected CAR T cell expansion) improved long-term leukemia clearance and PDX model ‘remission’ in both lower-dose and higher-dose TSLPRCART cohorts. Statistical analyses were performed for (**B**) and (**C**) with one-way ANOVA and Tukey post-test for multiple comparisons for surviving cohorts at day 56. *p < 0.05, **p < 0.01, ***p < 0.001, ****p < 0.0001.

We next sought to ascertain if a greater interval between TSLPRCART and initiation of JAKi could further minimize the observed direct antagonism upon early CAR T cell proliferation. Using the partially ruxolitinib-sensitive *CRLF2*-rearranged ALL121 PDX model (Fig. 4A and Supplementary Fig. 4), we detected robust initial activity of TSLPRCART at both low (1e6) and moderate (2.5e6) T cell doses. Higher TSLPRCART dosing (5e6) was not further pursued in the more delicate ALL PDX models given observed fatality and cytokine-associated hyperinflammation (Fig. 1A and not shown). However, xenograft mice in both TSLPRCART treatment groups subsequently died at approximately day 35 without detectable human ALL, but with robust T cell expansion and increasing IFN-γ levels (Fig. 4A–C, orange), potentially related to mortality from xenogeneic graft-versus-host disease (GVHD) [42]. Importantly, premature animal death in leukemia remission was ameliorated by co-administration of ruxolitinib with best anti-leukemia activity and animal survival seen with ruxolitinib introduction at day 14 post-TSLPRCART (Fig. 4A–C, purple). As in prior experiments, ruxolitinib co-administration resulted in reduced TSLPRCART and IFN-γ levels in the peripheral blood, which both recovered over time following JAKi withdrawal (Fig. 4B, C). Taken together, these data suggest that unhindered activation and expansion of TSLPRCART during an initial two-week period was necessary to preserve desired CAR T cell activity in vivo in *CRLF2*-R ALL xenograft mice and that subsequent ruxolitinib co-administration could be used both to dampen inflammatory toxicity and to sustain longer-term leukemia remission.

### Ruxolitinib maintenance therapy after TSLPRCART prevents Ph-like ALL relapse and reduces TSLPRCART phenotypic exhaustion

**Fig. 5:**
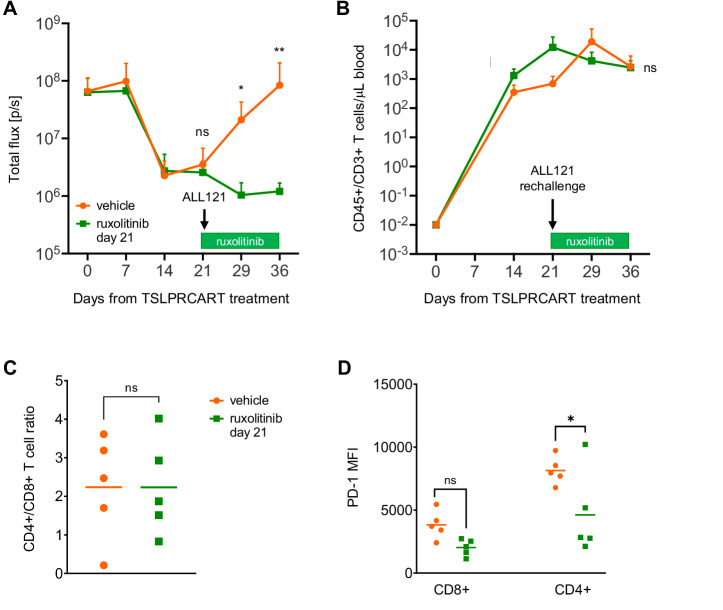
‘Maintenance’ therapy with ruxolitinib following TSLPRCART-induced ALL clearance prevents in vivo leukemia relapse*.* **A** Luciferase-transduced *IGH::CRLF2*/*JAK2*^R683G^-mutant ALL121 PDX model cells (1e6) were injected IV in NSG mice. Once engraftment was documented by BLI, all mice (*n* = 10) were treated IV with 2.5e6 TSLPRCART and followed by weekly BLI measurements. After documentation of TSLPRCART-induced leukemia clearance, mice were rechallenged IV with 1e7 ALL121 cells, and cohorts of 5 mice were randomized at day 21 to continued receipt of control chow (orange) or new administration of ruxolitinib chow (green) *ad libitum* for 2 weeks (horizontal green bar). Mice co-treated with ruxolitinib at ALL121 rechallenge remained in remission, while control chow-fed mice experienced leukemia progression, as assessed by BLI. **B** Weekly flow cytometric quantification of human CD3+/CD45+ T cells in murine peripheral blood showed no reduction of TSLPRCART numbers in ruxolitinib- (green) versus control-treated (orange) mice or in **(C)** the CD4:CD8 ratio of T cells harvested from end-study murine spleens at day 36. **D** Ruxolitinib treatment significantly decreased surface expression of the exhaustion marker PD-1 in CD4+ and CD8+ T cell subsets is reported as median fluorescence intensity (MFI) measured by flow cytometric analyses. PD-1 expression on TSLPRCART is decreased with late ruxolitinib co-treatment. After data normality assessment, statistical analyses were performed for (**A**) and (**B**) by unpaired Mann-Whitney tests at each timepoint, (**C**) with an unpaired *t*-test, and (**D**) with one-way ANOVA and Šidák post-test for multiple comparisons between vehicle and ruxolitinib conditions for CD4+ and CD8+ subpopulations. ns not significant, *p < 0.05, **p < 0.01.

We next postulated that sequenced ruxolitinib exposure could also improve persisting TSLPRCART fitness and decrease exhaustion. To test this hypothesis, we performed leukemia re-challenge experiments in our ALL121 xenografts to model relapse. Engrafted PDX mice were treated with TSLPRCART (2.5e6) to induce leukemia remission, then re-challenged 21 days later with TSLPR+/CRLF2+ALL121 cells without or with concomitant ruxolitinib treatment. Leukemia-rechallenged mice treated with vehicle (no ruxolitinib) promptly demonstrated detectable ALL progression and died within 2 weeks (Fig. 5A, orange), implying diminished functionality of residual detectable TSLPRCART. Conversely, ruxolitinib treatment of leukemia-rechallenged mice resulted in ALL re-control (Fig. 5A, green), suggesting that previously-activated/expanded CAR T cells were less sensitive to potentially deleterious direct effects of JAKi. Importantly, ruxolitinib addition at day 21 did not alter TSLPRCART re-expansion in murine peripheral blood (Fig. 5B). At end-study, no difference in the TSLPRCART CD4+/CD8+ ratio was detected in murine spleens from the ruxolitinib versus vehicle co-treatment cohorts (Fig. 5C), although surface expression of PD-1 was significantly decreased in CD4+ T cells exposed to ruxolitinib (Fig. 5D, green versus orange) without changes in surface expression of activation marker CD25 or of intracellular IFN-γ or IL-2 (Supplementary Fig. 5) following ALL121 rechallenge. These data show that a staggered JAKi maintenance therapy-type approach may alter T cell activation and exhaustion characteristics, improve longer-term CAR T cell anti-leukemia activity, and prevent CRLF2+ ALL relapse following TSLPRCART immunotherapy.

### TSLPRCART and ruxolitinib co-therapy is effective against *CRLF2*-rearranged Down syndrome-associated ALL

**Fig. 6:**
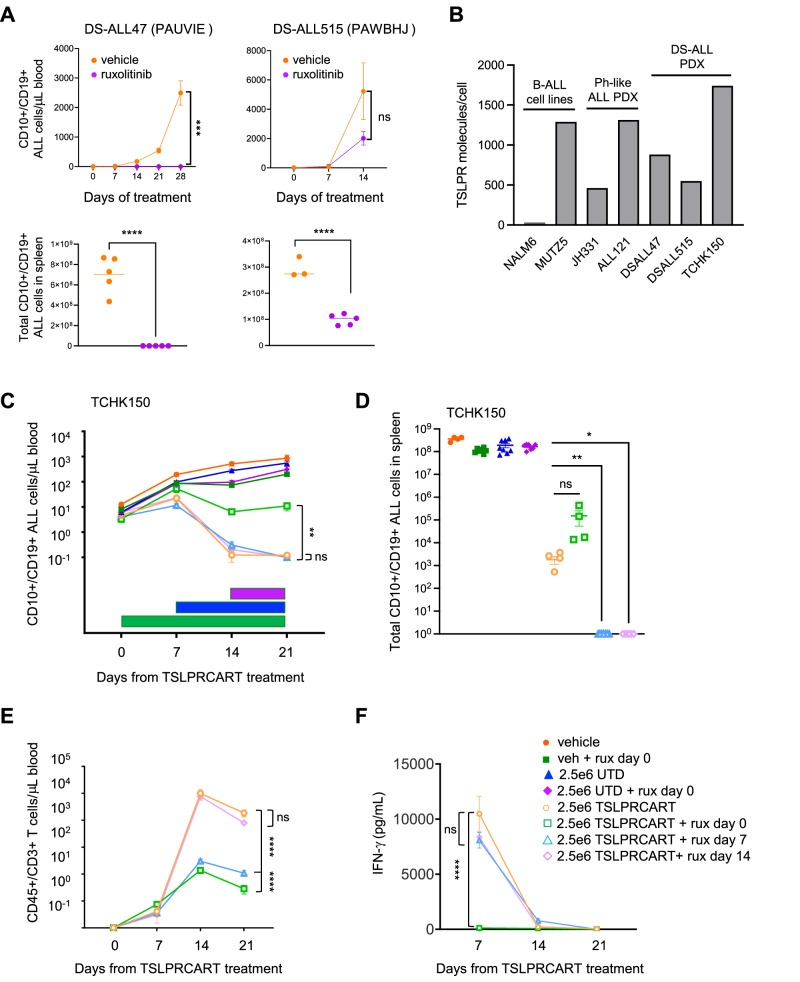
In vivo ruxolitinib and TSLPRCART sensitivity of *CRLF2*-rearranged Ph-like ALL is recapitulated in Down syndrome-associated ALL. **A** NSG mice were engrafted with 5e5 DSALL47 (left) or 1e6 DSALL515 (right) PDX model cells. Once >1% CD10+/CD19+ human ALL cells were detectable in murine peripheral blood, cohorts of 5 mice were randomized to treatment with control (orange) or ruxolitinib (purple) chow *ad libitum*. Leukemia burden was monitored weekly by quantitative flow cytometric analysis of human CD10+/CD19+ALL cells in peripheral blood (top panels) and in end-study spleens (bottom panels), which was determined by rate of leukemia progression in control mice for each model. Significant reduction of DS-ALL cell numbers in peripheral blood and spleens was detected in both tested models with near-curative effect after 4 weeks of ruxolitinib treatment. **B** Flow cytometric quantification of TSLPR surface antigen density demonstrates similar ranges of expression in *CRLF2*-rearranged Ph-like and DS-ALL PDX models, suggesting similar potential for therapeutic activity of TSLPRCART. *CRLF2* wild-type NALM-6 and *CRLF2-*rearranged MUTZ5 ALL cell lines were used as negative and positive controls, respectively. **C** NSG mice were engrafted with 1e6 TCHK150 ALL PDX model cells. Once >1% CD10+/CD19+ human ALL cells were detectable in murine peripheral blood, cohorts of 5 mice were randomized to IV treatment with saline, 2.5e6 UTD, or 2.5e6 TSLPRCART. Additional cohorts of TSLPRCART-treated mice (*n* = 5) were also randomized to simultaneous (day 0, green bar) or delayed (day 7, blue bar or day 14, purple bar) administration of ruxolitinib chow *ad libitum*. Animals were monitored weekly by quantitative flow cytometry analysis of human CD45+/CD10+/CD19+ ALL cells in murine peripheral blood and (**D**) end-study spleens. Delayed ruxolitinib co-treatment with TSLPRCART improved leukemia clearance compared to monotherapy or simultaneous co-treatment in this DS-ALL model. TSLPR surface expression remained unchanged by quantitative flow cytometric analysis in residual ALL cells in TSLPRCART-treated animals where applicable (data not shown). **E** Flow cytometric quantification of CD45+/CD3+ T cells in murine peripheral blood demonstrated no inhibition of TSLPRCART proliferation in vivo with day 14 ruxolitinib co-administration (lavender) compared to TSLPRCART monotherapy (light orange), whereas day 0 (light green) and day 7 (light blue) ruxolitinib exposure significantly impaired T cell numbers. **F** ELISA was performed to quantify human IFN-γ in murine plasma prepared from weekly peripheral venous blood. Significant dampening of IFN-γ production was detected with TSLPRCART and day 0 ruxolitinib co-treatment compared to TSLPRCART monotherapy (light green versus light orange). Statistical analyses were performed for (**A**) with unpaired *t*-tests and for (**C**), (**D**), (**E**), and (**F**) with 2-way ANOVA/mixed effects analysis and Dunnett post-test for multiple comparisons using the TSLPRCART condition as the comparator. ns not significant, *p < 0.05, **p < 0.01, ****p < 0.0001.

Given the high frequency of *CRLF2* rearrangements and *JAK2* point mutations in children and AYAs with DS-ALL [8], we then tested the hypothesis that the sequenced TSLPRCART and ruxolitinib strategy would be efficacious in newly-created preclinical CRLF2 + DS-ALL models. We first demonstrated significant in vivo sensitivity of two *P2RY8::CRLF2*/*JAK2*^R683^-mutant (DS-ALL47) and *P2RY8::CRLF2*/*JAK2* wild-type (DS-ALL515) DS-ALL PDX models to ruxolitinib monotherapy (Fig. 6A). We also noted that *CRLF2*-rearranged DS-ALL cells from PDX models express similarly high levels of cell surface CRLF2/TSLPR to those of *CRLF2*-rearranged Ph-like ALL, as assessed by quantitative flow cytometry (Fig. 6B).

TSLPRCART treatment of a third *IGH::CRLF2*/*JAK2* wild-type DS-ALL PDX model (TCHK150) potently inhibited in vivo leukemia proliferation with impaired T cell functionality with simultaneous ruxolitinib co-administration at day 0. However, complete human ALL eradication in end-study murine spleens was achieved when ruxolitinib was added at day 7 or day 14 following TSLPRCART administration (Fig. 6C, D). Importantly, day 14-delayed ruxolitinib addition did not inhibit TSLPRCART expansion or human IFN-γ levels in murine plasma at subsequent timepoints when compared to TSLPRCART alone, which contrasted with observed suppression of T cell functionality with earlier ruxolitinib addition (Fig. 6E, F) and was consistent with results in Ph-like ALL models. These results were validated in additional experiments using the aforementioned DS-ALL515 PDX model. Interestingly, flow cytometric analysis of CD3+-gated TSLPRCART in end-study murine spleens revealed a decreased CD4:CD8 ratio in animals treated with simultaneous ruxolitinib (day 0 timepoint) and equivalent ratio at day 14-delayed ruxolitinib to that of TSLPRCART alone (Supplementary Fig. 6).

## Discussion

Patients with Ph-like ALL continue to have unacceptably high relapse rates despite best-available intensive multi-agent chemotherapy regimens. Children and AYAs with DS-ALL also have high rates of chemotherapy-associated toxicity and inferior survival compared to their non-DS counterparts. These populations have accordingly been enriched in clinical trials of CD19- and CD22-targeted immunotherapies that have been highly successful in initial remission induction, but curative in the long-term for only a subset of patients given emergence of immunotherapeutic resistance caused by a variety of mechanisms. Analogous to the paradigm of non-cross-resistant cytotoxic chemotherapy regimens required to achieve cure in patients with ALL, we aimed in this preclinical study to co-attack essential CRLF2/TSLPR biology in Ph-like and DS-ALL via (1) unique cell surface antigen-targeted CAR T cell immunotherapy and (2) intracellular constitutive signaling-targeted tyrosine kinase inhibition.

We previously developed TSLPRCART immunotherapy and reported potent preclinical in vitro and in vivo anti-leukemia activity in *CRLF2*-ovexpressing ALL cell lines and xenograft models [35]. With more detailed in vivo testing in additional Ph-like and DS-ALL PDX models in the present work, we observed that higher doses of TSLPRCART needed for complete ALL eradication in some models also induced inflammatory cytokine-mediated toxicity and animal mortality. While we hypothesized that addition of the JAK1/2 inhibitor ruxolitinib to TSLPRCART would have synergistic efficacy against *CRLF2*-R ALL, we instead observed that simultaneous co-administration of ruxolitinib blunted TSLPRCART cytokine production and expansion, leading to suboptimal leukemia clearance. This phenomenon was not entirely surprising given the important role of JAK signaling in normal T cell functionality. However, our dual therapeutic strategy also beneficially protected against CAR T cell-induced toxicity and ultimately improved long-term survival of in vivo animal models. Importantly, we observed that JAKi-mediated suppression of TSLPRCART proliferation and anti-leukemia activity was reversible upon ruxolitinib withdrawal. Our subsequent experiments with iteratively-sequenced co-therapy convincingly demonstrate that delaying ruxolitinib exposure until after peak activation and expansion of TSLPRCART markedly improved desired in vivo anti-leukemia activity and reduced inflammation. Our data showing complete recovery of TSLPRCART functionality after ruxolitinib removal, including the ability to clear a CRLF2+ ALL relapse challenge, suggest that ruxolitinib maintenance therapy following TSLPRCART-induced leukemia remission could potentially prevent Ph-like and DS-ALL relapse while promoting long-term TSLPRCART persistence and remission durability and perhaps also minimizing antigen-loss relapse via antigen-independent targeting of critical intracellular signaling dependencies.

CRS is the most common inflammatory toxicity following CAR T cell immunotherapy. Current clinical management consensus [31] includes blockade of pathologic inflammatory cytokines via tocilizumab (anti-interleukin-6 receptor monoclonal antibody) [31], emapalumab (anti-interferon-γ monoclonal antibody) [45], anakinra (interleukin-1 receptor antagonist) [46], and/or corticosteroids, which may confer potential detrimental immunomodulatory effects on CAR T cell efficacy with extended use. Strategies for direct modulation of T cells to minimize their excessive inflammatory cytokine production upon target antigen contact have demonstrated promise in preclinical studies, including reversible dampening of normal endogenous T cell (needed for blinatumomab engagement) or CD19CART functionality when exposed in vitro to the SRC/ABL inhibitor dasatinib and protection from CRS in B-ALL models [47, 48]. Clinical anecdotes have reported successful mitigation of life-threatening CRS via dasatinib administration to CAR T cell-treated patients. Some clinical trials are now also incorporating dasatinib into ex vivo manufacturing processes with a goal of decreasing T cell exhaustion that may lead to poor long-term CAR T cell persistence and increased re-relapse risk in patients. In pilot studies, we also explored and compared potential in vitro effects of ruxolitinib and dasatinib upon our CAR T cell immunotherapies. We interestingly observed similarly reduced expression of T cell activation (CD25, CD71) and exhaustion (PD-1, LAG3) markers in CD3/CD28 bead-stimulated CD19CART or TSLPRCART co-incubated with ruxolitinib or dasatinib versus non-TKI controls (Supplementary Fig. 7A, B). CAR T cell-mediated production of IFN-γ with target antigen-positive non-Ph-like and Ph-like ALL cell line (eg, CD19+/TSLPR- NALM-6, CD19+/TSLPR+MUTZ5, CD19+/TSLPR- TVA-1 [49]) co-incubation was also analogously suppressed in the presence of concomitant dasatinib or ruxolitinib compared to no TKI exposure (Supplementary Fig. 7C). These preliminary data suggest that ruxolitinib has comparable effects upon CAR T cell functionality and phenotype to those of dasatinib, but more detailed studies are needed for validation.

Ruxolitinib is approved for treatment of patients with GVHD after allogeneic hematopoietic stem cell transplantation [36] and is under investigation for other hyperinflammatory diseases, such as hemophagocytic lymphohistiocytosis [37]. Since the initiation of our preclinical studies testing the hypothesis that JAKi and TSLPRCART co-therapy would have synergistic anti-leukemia activity against *CRLF2*-rearranged ALL [38, 39], a few case descriptions have also described successful use of ruxolitinib in patients with tocilizumab- and steroid-refractory CD19CART-induced CRS with decreases in detectable serum cytokine levels following administration of ruxolitinib and clinical resolution of hyperinflammatory signs and symptoms [50, 51]. Correlative biology studies from these patients further show reversible dampening of CAR T cell expansion and cytotoxicity upon in vitro JAKi exposure [51, 52], concordant with observations from our preclinical studies of ruxolitinib co-treatment and subsequent withdrawal. Emerging data from a phase 2 clinical trial of adult patients with relapsed/refractory B-cell lymphomas treated with axicabtagene ciloleucel (CD19CART immunotherapy) has further demonstrated reduced frequency and severity of inflammatory sequelae in patients randomized to co-treatment with the selective JAK1 inhibitor itacitinib as CRS prophylaxis compared to placebo control [53].

JAK/STAT signaling is known to regulate a number of processes in both CD4+ and CD8+ T cell biology, including differentiation, expansion, and maintenance of homeostasis, predominantly via effects upon cytokine receptor signaling [54]. To our knowledge, our study is the first to report a more prominent ruxolitinib-mediated inhibition of CD4+ CAR T cells compared to CD8+ cells. Interestingly, emerging work has implicated a preferential role of CD4+ T cells, but not CD8+ T cells, in the pathogenesis of CRS [55, 56], which is congruent with our observations of ruxolitinib-mediated prevention of life-threatening cytokine-associated toxicities in TSLPRCART-treated animal models.

In summary, we demonstrate robust curative potential of TSLPRCART immunotherapy in preclinical models of human *CRLF2*-rearranged Ph-like ALL and DS-ALL and an optimized time-sequenced JAK inhibitor co-therapy strategy that both mitigates acute CRS-like toxicity and potential CAR T cell exhaustion and maximizes long-term leukemia remission durability. With analogous protective effects of ruxolitinib (and dasatinib) also detected in CD19CART experiments, we posit that this tactic may be broadly applicable to management of severe inflammatory toxicities induced by antibody-mediated or cellular immunotherapies.

Limitations of our study include an incomplete ability to assess potential on-target/off-tumor or off-target effects of ruxolitinib and TSLPRCART in immunocompromised mouse models, although immunohistochemical analyses in our original preclinical study detected weak or absent TSLPR staining of multiple normal tissues [35]. Future studies in syngeneic murine models of B-ALL treated with CAR T cells [57] and ruxolitinib could potentially help to address this knowledge gap. It is unlikely that ruxolitinib will cause untoward clinical toxicity given broad experience to date in patients with myeloproliferative neoplasms [58], ALL [21], and/or GVHD [36] treated with JAK inhibitors without or with multi-agent chemotherapy, but is plausible that ruxolitinib combination with CAR T cell immunotherapies could induce deleterious clinical effects or new resistance mechanisms not predicted from preclinical model studies. Clinical investigation is required to answer such questions.

To this end, a soon-to-open phase 1 trial based upon our preclinical data will study the safety and activity of TSLPRCART immunotherapy in children and AYAs with relapsed/refractory CRLF2+ leukemias, including Ph-like ALL and DS-ALL. This trial will help us to understand both therapeutic and toxicity potentials of targeting the uniquely highly-expressed TSLPR surface antigen in patients with these high-risk leukemias and will facilitate identification of biomarkers of treatment response or failure. If TSLPRCART monotherapy-induced remissions are observed in treated subjects with Ph-like or DS-ALL, but subsequent relapses occur, next-generation clinical studies could investigate a time-sequenced ruxolitinib co-therapy maintenance strategy or bispecific antigen targeting [40, 59, 60]. Formal evaluation of JAK inhibition in patients with severe steroid-refractory inflammatory toxicities following CD22CART [61, 62], CD19CART, or other CAR T cell immunotherapies may also be more broadly warranted.

## Supplementary information

Supplementary Material

Supplementary Figures

## Data Availability

Human ALL cell lines used in these studies are publicly available via commercial sources. ALL PDX models may be made available from the authors upon written request and institutional approval of a material transfer agreement. The authors are glad to share guidance regarding protocols and assays used in these studies upon written request.
