# Supplementary material for: Co-targeting of the thymic stromal lymphopoietin receptor to decrease immunotherapeutic resistance in CRLF2-rearranged Ph-like and Down syndrome acute lymphoblastic leukemia
Source: Leukemia. 2024 Dec 16;39(3):555–67. doi: 10.1038/s41375-024-02493-3 (PMC11879877; doi:10.1038/s41375-024-02493-3)
Supplement: Supplementary file 1 — Supplementary Material [file 41375_2024_2493_MOESM1_ESM.docx]

**SUPPLEMENTARY DATA**

**SUPPLEMENTARY METHODS**

*Lentiviral vector production and CAR T cell transduction*

Thymic stromal lymphopoietin receptor-redirected chimeric antigen receptor T cells (TSLPRCART) and CD19CART were produced using our previously published methodologies^1, 2^ and as described herein. TSLPR and CD19 CAR-encoding lentiviral supernatants were produced by transfection of the lenti-X 293T cell line with plasmids encoding the CAR construct an retroviral packaging and envelope DNA (pMDLg/pRRE, pMD.2G, pRSV-Rev) as previously described.^1-3^ Supernatants were centrifuged at 5000 rpm for 10 minutes to remove cell debris, then stored at −80°C.

Human peripheral blood mononuclear cells from anonymous normal healthy donors were obtained from the University of Colorado Anschutz Medical Center. T cells were isolated with EasySep Human T cell isolation kit (StemCell Technologies; Cambridge, Massachusetts) and activated with a 1:1 ratio of CD3/CD28 Dynabeads (Gibco; Norristown, Pennsylvania) for 48 hours in AIM-V media containing 40 IU/mL recombinant IL-2 (Roche). Activated T cells were resuspended at 1 million cells in 1 mL of lentiviral supernatant plus 1 mL of fresh AIM-V media with 10 μg/mL protamine sulfate and 100 IU/mL IL-2 for *in vitro* culture in 6-well plates. Cells were centrifuged at 800g for 2 hours at 32°C and then incubated overnight at 37°C with 10% CO_2_. The CD3/CD28 beads were removed, and T cells were continued in expansion culture in AIM-V media containing 100 IU/mL IL-2. Fresh IL2-containing media was changed every 2 to 3 days until CAR T cell harvest on day 9. ‘Mock-transduced’ T cells as a negative control were created in parallel by omission of the CAR-containing plasmid from the lentiviral production.

*Additional Ph-like ALL cell lines*

CD19+/TSLPR- Ph-like ALL TVA-1 cells with *ETV6::ABL1* fusion were immortalized as *in vitro* cell line from a PDX model established from a primary patient sample (COG USI PAUXZX^4, 5^) by the laboratory of Dr David Fruman at the University of California, Irvine.^6^ TVA-1 cells were cultured for no longer than 2 months in RPMI medium containing 10% heat-inactivated fetal bovine serum, 2 mM L-glutamine and 100 U/mL penicillin/streptomycin and regularly confirmed to be *Mycoplasma*-free. The known *ETV6::ABL1* fusion was confirmed by fluorescence *in situ* hybridization testing by the Division of Genomic Diagnostics laboratory at the Children’s Hospital of Philadelphia.

*In vitro functional analyses of CAR T cells*

*In vitro* cell viability and inflammatory cytokine production assays were performed as previously described.^2, 3^ Briefly, in vitro cytotoxicity was measured by luciferase-reporter assay with 20,000 cells/well of luciferase-expressing CD19+/TSLPR- NALM-6, CD19+/TSLPR+ MUTZ5, or CD19+/TSLPR- TVA-1 human B-ALL cells co-incubated with 1:15 effector:target ratio [TSLPRCART] or 1:5 effector:target ratio [CD19CART]. Each condition was plated in triplicate with vehicle (negative control) or ruxolitinib or dasatinib (LC Laboratories) exposure *in vitro* at concentrations and incubation times indicated in the figure legends. Cell culture supernatant was removed for cytokine detection by enzyme-linked immunoabsorbent assay ([ELISA], BioLegend; San Diego, California) according to the manufacturer’s protocol. Cell lysates were collected and analyzed via the luciferase assay system (Promega) per its provided protocol. Both ELISA and luciferase assay readouts were performed using a Synergy 2 multi-detection plate reader (BioTek; Winooski, Vermont).

TSLPRCART expansion in the presence of ruxolitinib was assayed in a re-stimulation culture experiment. TSLPRCART were co-incubated at 1:1 E:T with TSLPR+ target MUTZ cells. Every 3-4 days, cells were removed, and live TSLPRCART cells were quantified by flow cytometry. Remaining cells were re-stimulated 1:1 with MUTZ5 and fresh ruxolitinib containing media. On day 7, a subset of ruxolitinib-exposed TSLPRCART were replated in the absence of ruxolitinib (withdrawal condition). Each condition was plated in triplicate.

Flow cytometric analysis of TSLPRCART and CD19CART CD4+/CD8+ subsets and expression of T cell markers in presence of ruxolitinib or dasatinib was also performed after culturing CAR T cells with 40 IU/mL recombinant IL-2 alone or with 1:3 E:T CD3/CD28 Dynabeads for 3 days. Each condition was plated in triplicate.

*ALL patient-derived xenograft models*

Patient-derived xenograft (PDX) models of *CRLF2*-rearranged Ph-like ALL (JHH331 and ALL121) and *CRLF2*-rearranged DS-ALL (DSALL47, DSALL515, and TCHK150; **Table 1**) were established as previously described or newly-created using viably cryopreserved specimens banked in the Children’s Oncology Group, Children’s Hospital of Philadelphia, or Texas Children’s Hospital leukemia biorepositories under institutional review board-approved research protocols following obtainment of informed consent in accordance with the Declaration of Helsinki.^4, 5, 7^ Briefly, primary leukemia cells from peripheral blood or bone marrow were injected intravenously (IV) into nonobese diabetic/severe combined immunodeficient (NOD.Cg-Prkdcscid) Il2rgtm1wjl/SzJ (NSG) mice. Spleens collected from human ALL-engrafted primary xenograft mice were processed and reinjected to create secondary and tertiary xenograft models for experimental studies.

*In vivo analyses of TSLPRCART and ruxolitinib treatment in animal models*

Cell line or secondary or tertiary xenografted human ALL specimens were IV-injected into 6-8 week old female or male NSG mice as previously described.^1^ For luciferase-expressing ALL PDX models, assessment of initial engraftment and serial monitoring of leukemia burden were performed with bioluminescent imaging with IVIS Lumina Imaging System (PerkinElmer; Waltham, Massachusetts) and analyzed with Living Image V4.7.3 software (PerkinElmer) as described.^2, 3, 8^ ALL engraftment in non-luciferase expressing xenograft models was quantified by flow cytometry analysis, and treatment was initiated once ≥1% human ALL was detectable in peripheral blood. Animals were randomised to treatment with IV saline, mock-transduced T cells, or TSLPRCART at the doses and timing indicated in the figure legends. Ruxolitinib-infused chow (2g/kg, a gift from Matthew Stubbs at the Incyte Corporation; Wilmington, Delaware)^5, 7^ was provided to the corresponding cohorts of mice at the timepoints indicated in the figure legends. Investigators were not blinded to the group allocation. Leukemia progression was monitored by weekly retro-orbital venous blood sampling via quantitative flow cytometry analysis of CD45+/CD19+ or CD10+/CD19+ human ALL cells and CD45+/CD3+ T cells in peripheral blood and in end-study spleens. Plasma from murine peripheral blood was processed via centrifugation at 2000g for 15 min at 4° C and stored at -80° C for subsequent batched cytokine analyses.

*Flow cytometry analyses*

*In vitro* cell cycle analyses of T cells was performed with RNase-containing propidium iodide stain using a Beckman Coulter Cytoflex flow cytometer. Flow cytometry data were analysed in Cytobank (Beckman-Coulter; Brea, California) or FlowJo (TreeStar; Ashland, Oregon). Each condition was plated in triplicate.

Flow cytometry analysis of cell surface activation and exhaustion markers on CD19CART and TSLPRCART without or with *in vitro* ruxolitinib or dasatinib exposure was performed using human CD8-AF488 (Invitrogen #53-0081-82), CD4-APC (Invitrogen #17-0049-42), CD8-APC-Cy7 (Invitrogen #47-0087-42), CD25-AF488 (Invitrogen #11-0259-42), CD71-FITC (Invitrogen #11-0719-42), PD-1-PE (Invitrogen #12-9969-42), and/or PE-TSLPR (Invitrogen #12-5499-42) antibodies. Compensation was performed with UltraComp eBeads^TM^ Plus (Invitrogen #01333342). Flow cytometry data were captured using Cytoflex flow cytometer and analysed in FlowJo as above.

For *in vivo* animal studies, human ALL and T cells were measured weekly in murine peripheral blood and in end-study spleens by flow cytometry as previously described.^2, 5, 7, 9^ Briefly, blood or spleen samples underwent red blood cell lysis with TAC buffer followed by antibody staining with the following antibodies depending on the specific experiment: human CD45-APC (BioLegend #368512), CD19-PE (Invitrogen #12-0199-42), CD10-PE-Cy7 (BioLegend #982210), CD3-V450 (Invitrogen #48-0038-42), CD8-AF488, CD4-APC, CD8-APC-Cy7, CD25-AF488, CD71-FITC, PD-1-PE, and/or PE-TSLPR as above. CountBright beads (Invitrogen #C36995) were used for human ALL and T cell quantification. Intracellular cytokine staining with IL-2-PE (Invitrogen #12-7029-42) and IFN-γ-APC-Cy7 (Biolegend #502529) was performed with a Cytofix/Cytoperm Kit (BD Biosciences #554714; Franklin Lakes, New Jersey) according to the manufacturer’s recommendations. Compensation was performed with UltraComp eBeads^TM^ Plus. Flow cytometry data were captured using BD FACSVerse or Beckman Coulter Cytoflex flow cytometers and analysed via CytoBank or FlowJo as above.

*Single-cell multiplex secretome analyses*

TSLPRCART products were thawed and incubated in complete medium RPMI (Gibco) containing 10% fetal bovine serum (Cytiva; Marlborough, Massachusetts), 1x Glutamax (Gibco), 100U/mL penicillin-streptomycin (Gibco), and 40 IU/mL recombinant IL-2 (rhIL-2; Roche; Indianapolis, Indiana) for 24 hours overnight at 37°C, 5% CO2. After recovery, CD8+ and CD4+ T cell subsets were magnetically separated using CD8 microbeads (Miltenyi; Gaithersburg, Maryland). CD4+ and CD8+ T cells were then separately cocultured with TSLPR+ MUTZ5 ALL cells at a 1:2 ET ratio with or without addition of ruxolitinib 0.1 μM for 20h at 37°C in 5% CO2. Following incubation, MUTZ5 cells were magnetically depleted from the co-culture using a biotin-labeled anti-human CD19 antibody (Invitrogen #13-0199-82) conjugated with Dynabeads M-280 streptavidin (ThermoFisher Scientific). The remaining TSLPRCART were stained with membrane stain AF647 (Isoplexis; Branford, Connecticut). Finally, 30,000 viable cells were loaded onto the 32-plex human single-cell IsoCode-Human Adaptive Immune chips (IsoPlexis). Chips were then incubated in an IsoSpark machine for 16 hours for scanning, and data analysis was performed using the IsoSpeak software v.3.0.1 (IsoPlexis).

**SUPPLEMENTARY REFERENCES**

1. Qin H, Cho M, Haso W, Zhang L, Tasian SK, Oo HZ*, et al.* Eradication of B-ALL using chimeric antigen receptor-expressing T cells targeting the TSLPR oncoprotein. *Blood* 2015 Jul 30; **126**(5)**:** 629-639.

2. Niswander LM, Graff ZT, Chien CD, Chukinas JA, Meadows CA, Leach LC*, et al.* Potent preclinical activity of FLT3-directed chimeric antigen receptor T-cell immunotherapy against FLT3- mutant acute myeloid leukemia and KMT2A-rearranged acute lymphoblastic leukemia. *Haematologica* 2023 Feb 1; **108**(2)**:** 457-471.

3. Qin H, Yang L, Chukinas JA, Shah N, Tarun S, Pouzolles M*, et al.* Systematic preclinical evaluation of CD33-directed chimeric antigen receptor T cell immunotherapy for acute myeloid leukemia defines optimized construct design. *J Immunother Cancer* 2021 Sep; **9**(9).

4. Ding YY, Kim H, Madden K, Loftus JP, Chen GM, Allen DH*, et al.* Network Analysis Reveals Synergistic Genetic Dependencies for Rational Combination Therapy in Philadelphia Chromosome-Like Acute Lymphoblastic Leukemia. *Clin Cancer Res* 2021 Sep 15; **27**(18)**:** 5109-5122.

5. Hurtz C, Wertheim GB, Loftus JP, Blumenthal D, Lehman A, Li Y*, et al.* Oncogene-independent BCR-like signaling adaptation confers drug resistance in Ph-like ALL. *J Clin Invest* 2020 Jul 1; **130**(7)**:** 3637-3653.

6. Gotesman M, Vo T-T, Herzog L-O, Tea T, Mallya S, Tasian S*, et al.* mTOR inhibition enhances efficacy of dasatinib in ABL-rearranged Ph-like B-ALL. *Oncotarget* 2018 01/06; **9**.

7. Tasian SK, Teachey DT, Li Y, Shen F, Harvey RC, Chen IM*, et al.* Potent efficacy of combined PI3K/mTOR and JAK or ABL inhibition in murine xenograft models of Ph-like acute lymphoblastic leukemia. *Blood* 2017 Jan 12; **129**(2)**:** 177-187.

8. Tasian SK, Kenderian SS, Shen F, Ruella M, Shestova O, Kozlowski M*, et al.* Optimized depletion of chimeric antigen receptor T cells in murine xenograft models of human acute myeloid leukemia. *Blood* 2017 Apr 27; **129**(17)**:** 2395-2407.

9. Loftus JP, Yahiaoui A, Brown PA, Niswander LM, Bagashev A, Wang M*, et al.* Combinatorial efficacy of entospletinib and chemotherapy in patient-derived xenograft models of infant acute lymphoblastic leukemia. *Haematologica* 2021 Apr 1; **106**(4)**:** 1067-1078.

10. Rossi J, Paczkowski P, Shen YW, Morse K, Flynn B, Kaiser A*, et al.* Preinfusion polyfunctional anti-CD19 chimeric antigen receptor T cells are associated with clinical outcomes in NHL. *Blood* 2018 Aug 23; **132**(8)**:** 804-814.

**SUPPLEMENTARY FIGURES**

**Supplementary Figure 1**. ***In vitro* effects of ruxolitinib upon normal human donor T cells and CAR T cells. (A)** Western blot analysis of phosphorylated STAT5 (pSTAT5), phosphorylated ERK1/2 (pERK1/2), and β-actin in normal T cells from three different healthy donors cultured for 72 hours in absence (vehicle) or presence of ruxolitinib (0.1 or 0.5 µM). Blots were performed in triplicate (not shown). **(B)** Cell cycle analysis of healthy donor T cells (n=3) incubated with CD3/CD28 beads and vehicle (veh) or 0.5 µM ruxolitinib (rux) for 72 hours is summarized for total T cells and in the designated **(C)** CD4+ and  **(D)** CD8+ subsets. Depicted data represent the mean of three independent T cell donors plated in technical triplicates +/- standard error of the mean (SEM). **(E)** Luciferase-expressing *CRLF2* wild-type (TSLPR-negative) NALM-6 cells were seeded (20,000 cells) with or without CD19CART at 1:5 E (effector):T (target) cells ratio and treated with vehicle or ruxolitinib (0.1 and 0.5 μM). Cell viability via luciferase reporter assays (left) and production of IL-2 (middle) and IFN-γ (right) via ELISA were assayed at indicated time points. Depicted data represent the mean of technical triplicates +/- SEM. **(F)** Luciferase-expressing *CRLF2* wild-type NALM-6 cells were seeded (20,000 cells) with or without TSLPRCART at 1:5 E:T cells ratio and treated with vehicle or ruxolitinib (0.1 and 0.5 μM). Cell viability and cytokine production were assayed as in (E). Depicted data represent the mean of technical triplicates +/- SEM. After data normality assessment, statistical analyses were performed with Student t-test in (B), (C), and (D) and with one-way ANOVA and Tukey post-test for multiple comparisons in (E) and (F). *p<0.05, *** p<0.001, **** p<0.0001

**Supplementary Figure 2. Short-term ruxolitinib exposure decreases TSLPRCART polyfunctionality**. Single-cell proteomic analysis on a panel of secreted chemokines and cytokines was performed on TSLPRCART stimulated with CRLF2/TSLPR+ MUTZ5 cells in the presence of vehicle or ruxolitinib 0.5 μM for 20 hours. Polyfunctional single cells were defined as cells co-secreting at least two proteins. Secreted proteins were functionally categorized as effector, stimulatory, regulatory, chemoattractive, and inflammatory based upon their specific cytokine profiles as indicated. A polyfunctionality strength index (PSI) was defined as the percentage of polyfunctional cells multiplied by the signal intensity of the proteins secreted by those cells.^10^ PSI for CD4+ and CD8+ T cell subsets without and with ruxolitinib exposure is displayed.

**Supplementary Figure 3. Early *in vivo* ruxolitinib exposure decreases the anti-leukemia efficacy and cytokine production of TSLPRCART.** As described in Figure 3, mice engrafted with luciferase-expressing MUTZ5 were randomized to treatment with saline, 1e6 untransduced T cells (UTD), or lower-dose (1e6) or higher-dose (5e6) TSLPRCART. Ruxolitinib chow (rux) *ad libitum* was administered simultaneously at day 0 (green) or day 7 after T cell treatments (blue) and continued to day 42. Leukemia burden was measured weekly by BLI. The total flux measurements for bioluminescent imaging depicted in Figure 3A is displayed graphically for mice treated with **(A)** saline vehicle or UTD or **(B)** TSLPRCART at the indicated cell doses. (**C**) Human CD3+/CD45+ T cells were quantified weekly by flow cytometry analysis of peripheral blood of mice treated with UTD Plasma was collected at the indicated time points for IFN-γ quantification by ELISA assay in the **(D)** 1e6 TSLPRCART-treated and **(E)** 5e6 TSLPRCART-treated mice. Statistical analyses were performed by two-way ANOVA with Dunnett post-test for multiple comparisons using the vehicle control cohort (orange circle) as comparator in (A); all ruxolitinib-treated groups were significantly different at the day 42 timepoint, and UTD was not different from vehicle. The 1e6 TSLPRCART cohort (orange circle) was used as comparator in (B), and a significant difference for the 1e6 + ruxolitinib day 0 cohort was detected at day 42. Statistical analyses were performed with one-way ANOVA and Dunnett post-test for multiple comparisons using the vehicle control cohort (orange circle) as comparator in (C) and (D) with differences indicated for each time point where detected or are otherwise not significant. *p<0.05, **p<0.01, **** p<0.0001

**Supplementary Figure 4. Delayed JAK inhibitor co-treatment improves *in vivo* TSLPRCART activity against a ruxolitinib-sensitive *CRLF2*-rearranged Ph-like ALL PDX model.** As described in Figure 4, luciferase-transduced *IGH::CRLF2*/*JAK2*^R683G^-mutant ALL121 PDX model cells (1e6) were injected IV in NSG mice. Once engraftment was documented by BLI, cohorts of 5 mice were randomized to IV treatment with saline, 1e6 untransduced T cells (UTD), or lower-dose (1e6) or higher-dose (2.5e6) TSLPRCART. Ruxolitinib (rux) chow *ad libitum* was administered simultaneously at day 0 (green), day 7 (blue), or day 14 (purple) after T cell treatments and continued for 21 days in each cohort until days 21, 28, or 35, respectively. Leukemia burden was measured weekly by BLI with graphical display of total flux for mice treated with **(A)** saline vehicle or UTD, **(B)** 1e6 TSLPRCART, or **(C)** 5e6 TSLPRCART. (D) Human CD3+/CD45+ T cells were quantified weekly by flow cytometry analysis of peripheral blood of mice treated with lower-dose (left panel) or higher-dose (right panel) TSLPRCART Statistical analyses were performed with one-way ANOVA and Tukey post-test for multiple comparisons. *p<0.05, **p<0.01, ***p<0.001, **** p<0.0001

**Supplementary Figure 5. TSLPRCART characterization following “maintenance” *In vivo* ruxolitinib therapy**. As described in Figure 5, luciferase-transduced *IGH::CRLF2*/*JAK2*^R683G^-mutant ALL121 PDX model cells (1e6) were injected IV in NSG mice. Once engraftment was documented by BLI, all mice (n=10) were treated IV with 2.5e6 TSLPRCART and followed by weekly BLI measurements. After documentation of TSLPRCART-induced leukemia clearance, mice were rechallenged IV with 1e7 ALL121 cells, and cohorts of 5 mice were randomized at day 21 to continued receipt of control chow (orange) or new administration of ruxolitinib chow (green) *ad libitum* for 2 weeks. TSLPRCART cells in end study spleens at day 36. **(A)** Intracellular IFN- and **(B)** IL-2 and (**C**) surface expression of CD25 in CD4+ and CD8+ T cell subsets are reported as median fluorescence intensity (MFI) measured by quantitative flow cytometric analyses. Late ruxolitinib co-treatment did not alter IFN-γ, IL-2, or CD25 surface expression on CD4+ or CD8+ TSLPRCART cells, suggesting no difference in T cell activation status. After data normality assessment, statistical analyses were performed with a paired t-test, and no significant differences were detected.

**Supplementary Figure 6. *In vivo* ruxolitinib and TSLPRCART sensitivity of *CRLF2*-rearranged Ph-like ALL is recapitulated in Down syndrome-associated ALL.** NSG mice were engrafted with 1e6 DSALL515 PDX model cells. Once >1% CD10+/CD19+ human ALL cells were detectable in murine peripheral blood, cohorts of 5 mice were randomized to treatment with control (orange) or ruxolitinib chow *ad libitum* at day 0 (green), day 7 (blue), or day 14 (purple). Ruxolitinib was then withdrawn after 14 days of treatment for each relevant cohort. Leukemia burden was monitored weekly by quantitative flow cytometric analysis of **(A)** CD10+/CD19+ ALL cells and (**B)** human CD3+ T cells in peripheral blood and in end-study spleens. **(C)** ELISA was performed to quantify human IFN-γ in murine plasma prepared from weekly peripheral venous blood. Significant dampening of IFN-γ production was detected with 1e6 or 2.5e6 TSLPRCART and day 0 ruxolitinib co-treatment (dark green filled square or light green open square) compared to 1e6 or 2.5e6 TSLPRCART monotherapy (dark orange filled circle or light orange open circle), respectively, via one-way ANOVA with Tukey post-test for multiple comparisons. **(D)** Flow cytometric quantification of TSLPRCART CD4+ and CD8+ T cell subsets in murine spleens at day 28 demonstrated a significantly lower CD4:CD8 ratio in the day 0 ruxolitinib co-treatment cohort compared to day 14-delayed ruxolitinib cohort, as assessed by one-way ANOVA with Tukey post-test for multiple comparisons. *p<0.05, **** p<0.0001

**Supplementary Figure 7**. **Comparison of ruxolitinib and dasatinib *in vitro* effects upon CAR T cells.** (**A-B**) CD19CART and TSLPRCART were incubated with 40 IU/mL IL-2 in the absence (solid bars) or presence (striped bars) of CD3/CD28 Dynabeads in 1:3 E:T ratio for 96 hours with or without ruxolitinib rux 0.5 μM or dasatinib 10 nM. Surface expression of CD25, CD71, PD-1 and LAG-3 on CD4+ and CD8+ T cells was evaluated by flow cytometry analysis in (**A**) CD19CART and (**B**) TSLPRCART. Quantification of median fluorescent intensity (MFI) for CD4+ (top rows) and CD8+ T (bottom rows) cell subsets with technical triplicates for each condition is displayed +/- SEM. (**C**) MUTZ5, NALM-6 or TVA-1 cells were co-incubated *in vitro* with either TSLPRCART at 1:15 (E:T) ratio or CD19CART at 1:5 E:T in presence of vehicle or ruxolitinib at 0.1 and 0.5 μM concentrations or dasatinib at 1 and 10nM concentration. IFN-γ production via ELISA were measured at the indicated time points. Depicted data represent the mean of technical triplicates +/- SEM. After data normality assessment, statistical analyses were performed for (A, B and C) only between the beads-stimulated samples (striped bars) with one-way ANOVA with Tukey post-test for multiple comparisons and for (D) with one-way ANOVA and Dunnett post-test for multiple comparisons using the TSLPRCART or CD19CART condition as the comparator. Only significant differences are shown. *p<0.05, **p<0.01, ***p<0.001, ****p<0.0001.
