# Supplementary material for: Co-targeting of the thymic stromal lymphopoietin receptor to decrease immunotherapeutic resistance in CRLF2-rearranged Ph-like and Down syndrome acute lymphoblastic leukemia
Source: Leukemia. 2024 Dec 16;39(3):555–67. doi: 10.1038/s41375-024-02493-3 (PMC11879877; doi:10.1038/s41375-024-02493-3)
Supplement: Supplementary file 2 — Supplementary Figures [file 41375_2024_2493_MOESM2_ESM.pptx]

## Slide 1
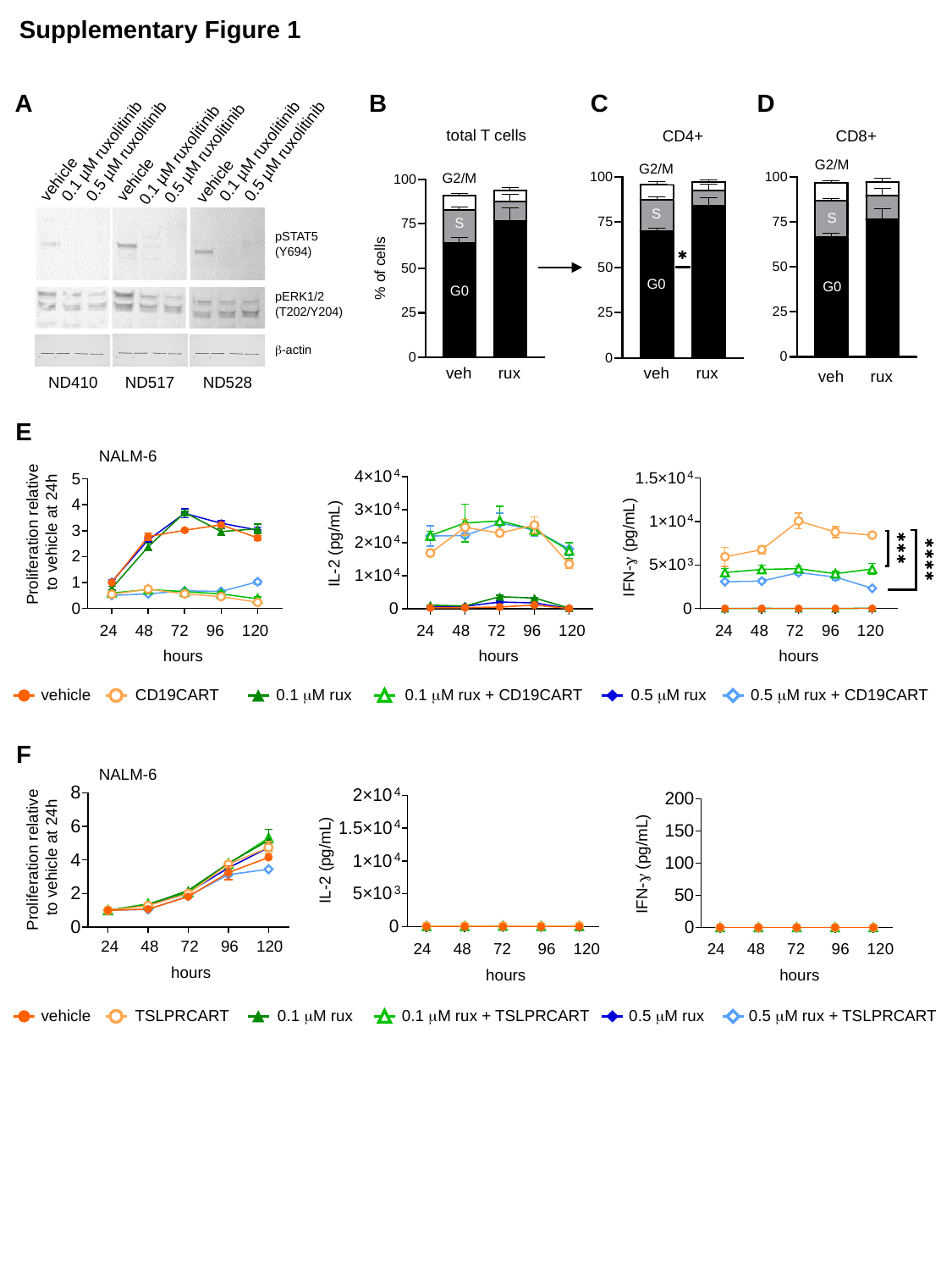

Supplementary Figure 1
0.1 µM ruxolitinib
0.5 µM ruxolitinib
0.1 µM ruxolitinib
0.1 µM ruxolitinib
0.5 µM ruxolitinib
0.5 µM ruxolitinib
vehicle
vehicle
vehicle
pSTAT5
(Y694)
pERK1/2
(T202/Y204)
b-actin
ND410
ND517
ND528
A
B
C
D
CCD4+D4+
CD4+
CD8+
G2/M
S
G0
total T cells
G2/M
G2/M
S
S
G0
G0
veh rux
veh rux
veh rux
E
NALM-6
Proliferation relative
to vehicle at 24h
IL-2 (pg/mL)
IFN-g (pg/mL)
24 48 72 96 120
24 48 72 96 120
24 48 72 96 120
hours
hours
hours
vehicle CD19CART 0.1 mM rux 0.1 mM rux + CD19CART 0.5 mM rux 0.5 mM rux + CD19CART
F
NALM-6
Proliferation relative
to vehicle at 24h
IL-2 (pg/mL)
IFN-g (pg/mL)
24 48 72 96 120
hours
24 48 72 96 120
hours
24 48 72 96 120
hours
vehicle TSLPRCART 0.1 mM rux 0.1 mM rux + TSLPRCART 0.5 mM rux 0.5 mM rux + TSLPRCART

## Slide 2
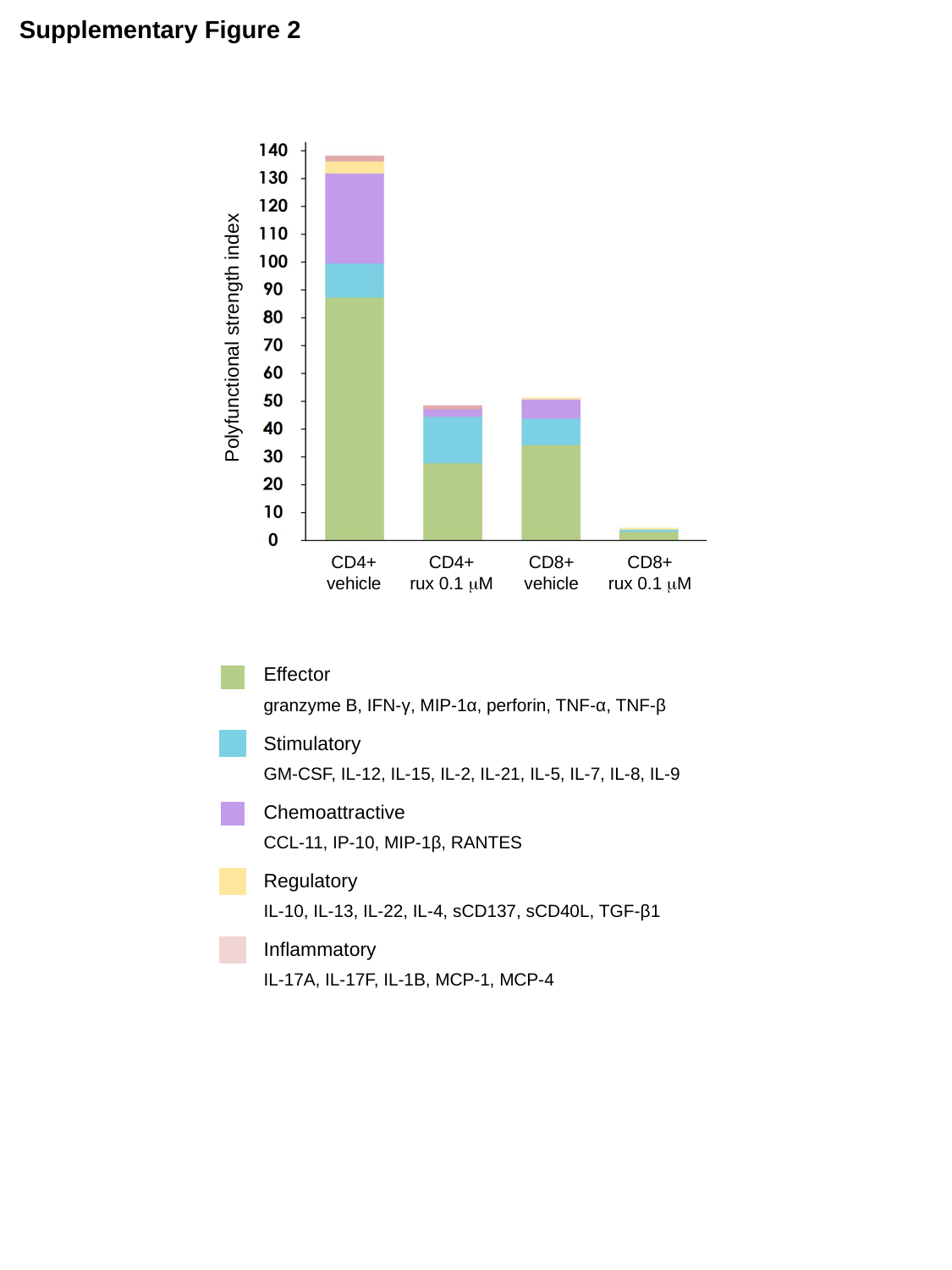

Supplementary Figure 2
Polyfunctional strength index
CD4+
vehicle
CD4+
rux 0.1 mM
CD8+
vehicle
CD8+
rux 0.1 mM
Effector
granzyme B, IFN-γ, MIP-1α, perforin, TNF-α, TNF-β
Stimulatory
GM-CSF, IL-12, IL-15, IL-2, IL-21, IL-5, IL-7, IL-8, IL-9
Chemoattractive
CCL-11, IP-10, MIP-1β, RANTES
Regulatory
IL-10, IL-13, IL-22, IL-4, sCD137, sCD40L, TGF-β1
Inflammatory
IL-17A, IL-17F, IL-1B, MCP-1, MCP-4

## Slide 3
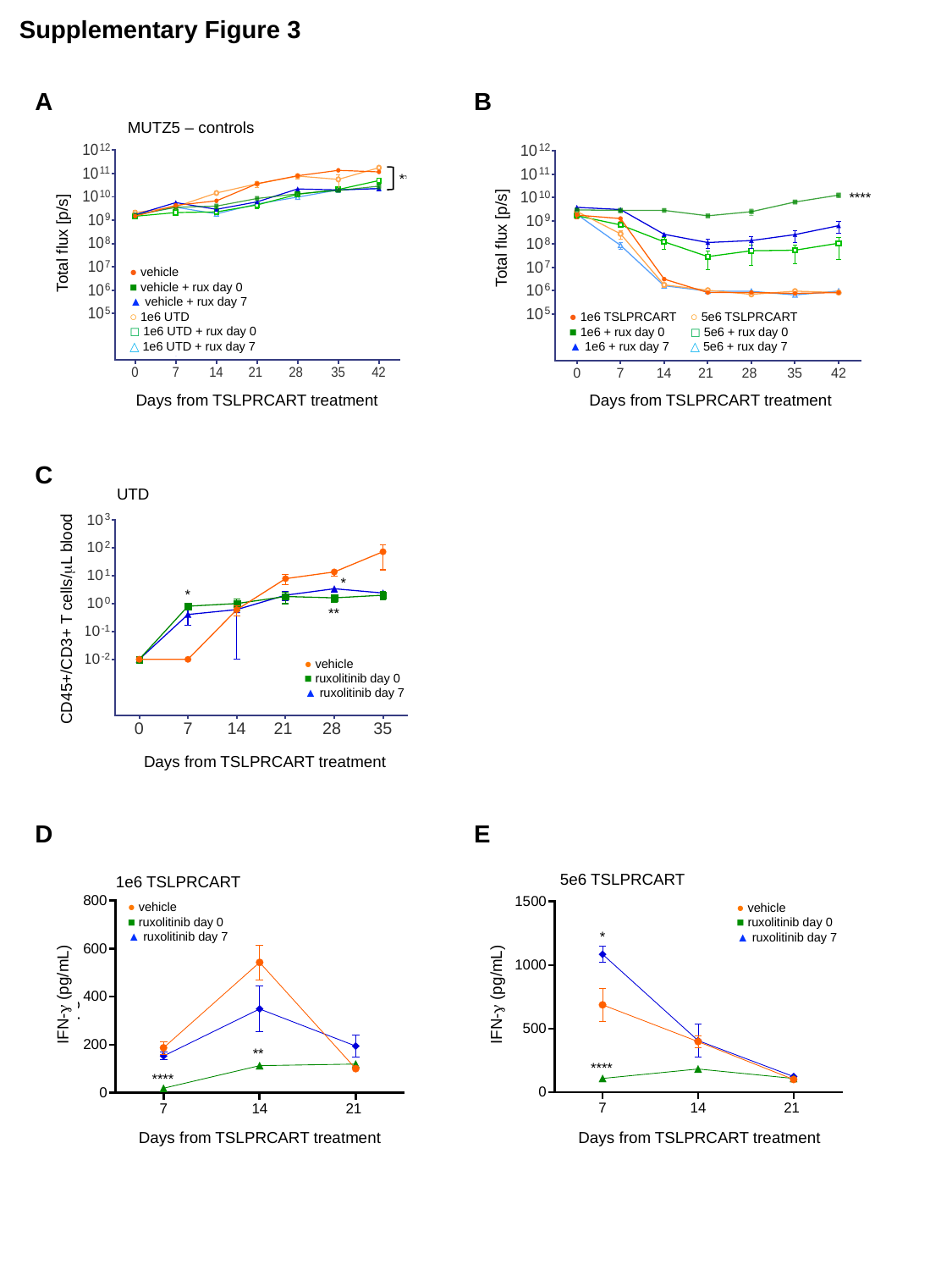

Supplementary Figure 3
A
B
MUTZ5 – controls
Total flux [p/s]
Total flux [p/s]
● vehicle
■︎ vehicle + rux day 0
▲ vehicle + rux day 7
○ 1e6 UTD
◻︎ 1e6 UTD + rux day 0
△ 1e6 UTD + rux day 7
● 1e6 TSLPRCART
■︎ 1e6 + rux day 0
▲ 1e6 + rux day 7
○ 5e6 TSLPRCART
◻︎ 5e6 + rux day 0
△ 5e6 + rux day 7
Days from TSLPRCART treatment
Days from TSLPRCART treatment
E
D
5e6 TSLPRCART
1e6 TSLPRCART
● vehicle
■ ruxolitinib day 0
▲ ruxolitinib day 7
IFN-g (pg/mL)
IFN-g (pg/mL)
Days from TSLPRCART treatment
Days from TSLPRCART treatment
****
● vehicle
■ ruxolitinib day 0
▲ ruxolitinib day 7
*
**
****
****
**
C
UTD
● vehicle
■ ruxolitinib day 0
▲ ruxolitinib day 7
*
*
**
CD45+/CD3+ T cells/mL blood
Days from TSLPRCART treatment

## Slide 4
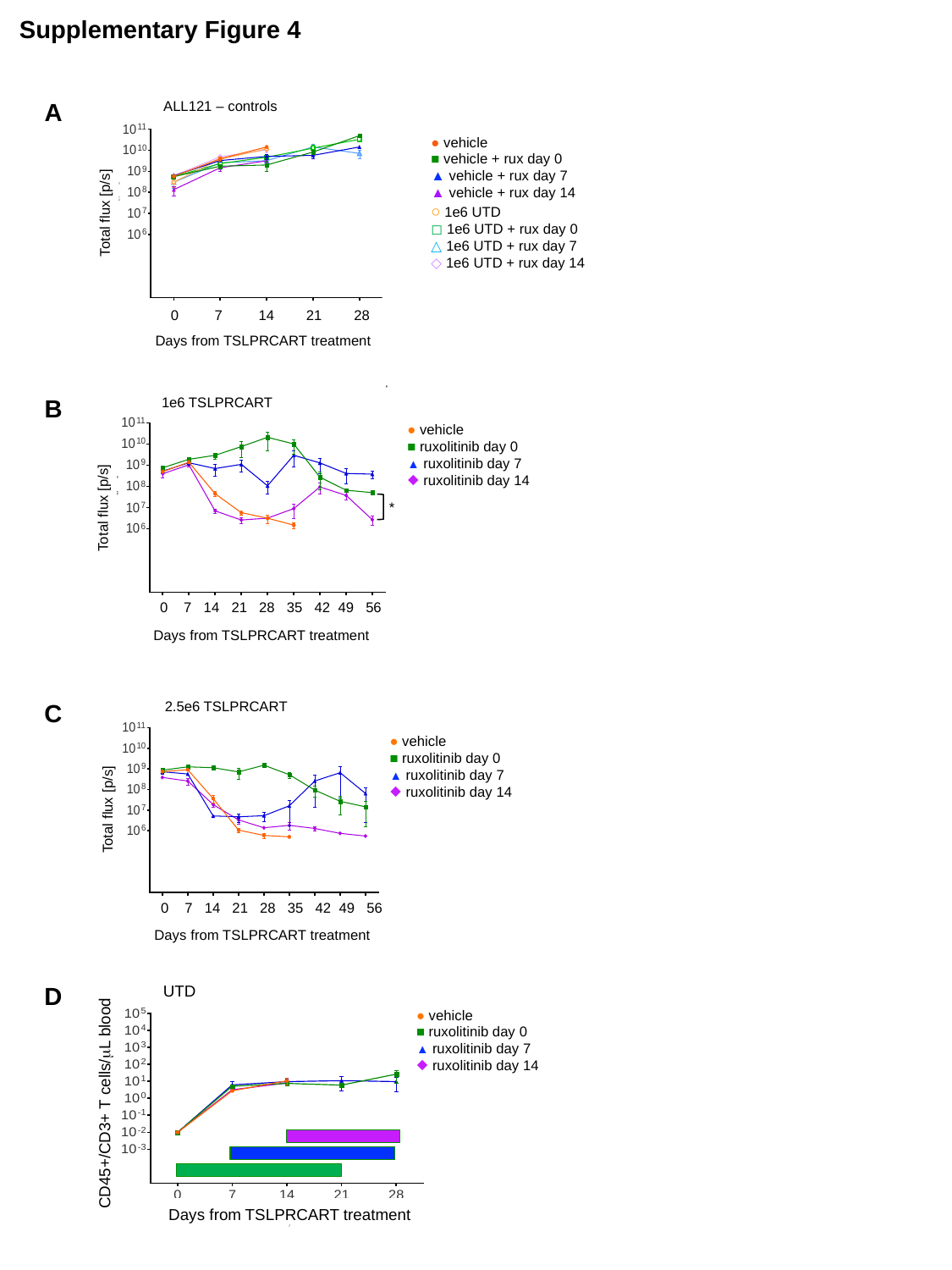

Supplementary Figure 4
A
ALL121 – controls
● vehicle
■︎ vehicle + rux day 0
▲ vehicle + rux day 7
▲ vehicle + rux day 14
○ 1e6 UTD
◻︎ 1e6 UTD + rux day 0
△ 1e6 UTD + rux day 7
◇ 1e6 UTD + rux day 14
Total flux [p/s]
 0 7 14 21 28
Days from TSLPRCART treatment
B
1e6 TSLPRCART
● vehicle
■ ruxolitinib day 0
▲ ruxolitinib day 7
⬥ ruxolitinib day 14
*
Total flux [p/s]
0 7 14 21 28 35 42 49 56
Days from TSLPRCART treatment
C
2.5e6 TSLPRCART
● vehicle
■ ruxolitinib day 0
▲ ruxolitinib day 7
⬥ ruxolitinib day 14
Total flux [p/s]
0 7 14 21 28 35 42 49 56
Days from TSLPRCART treatment
D
UTD
● vehicle
■ ruxolitinib day 0
▲ ruxolitinib day 7
⬥ ruxolitinib day 14
CD45+/CD3+ T cells/mL blood
Days from TSLPRCART treatment
/

## Slide 5
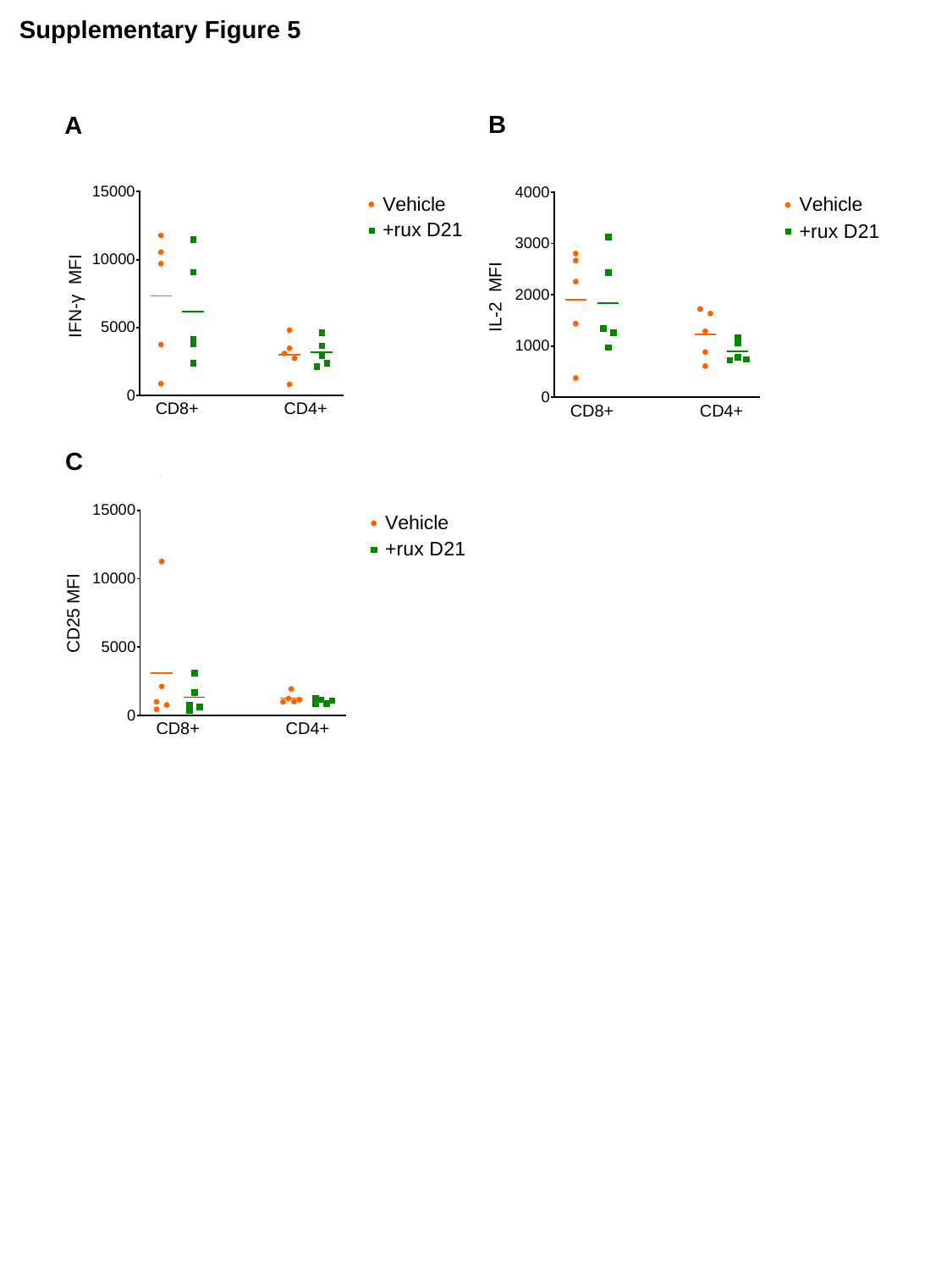

Supplementary Figure 5
B
A
IFN-γ MFI
IL-2 MFI
C
CD25 MFI

## Slide 6
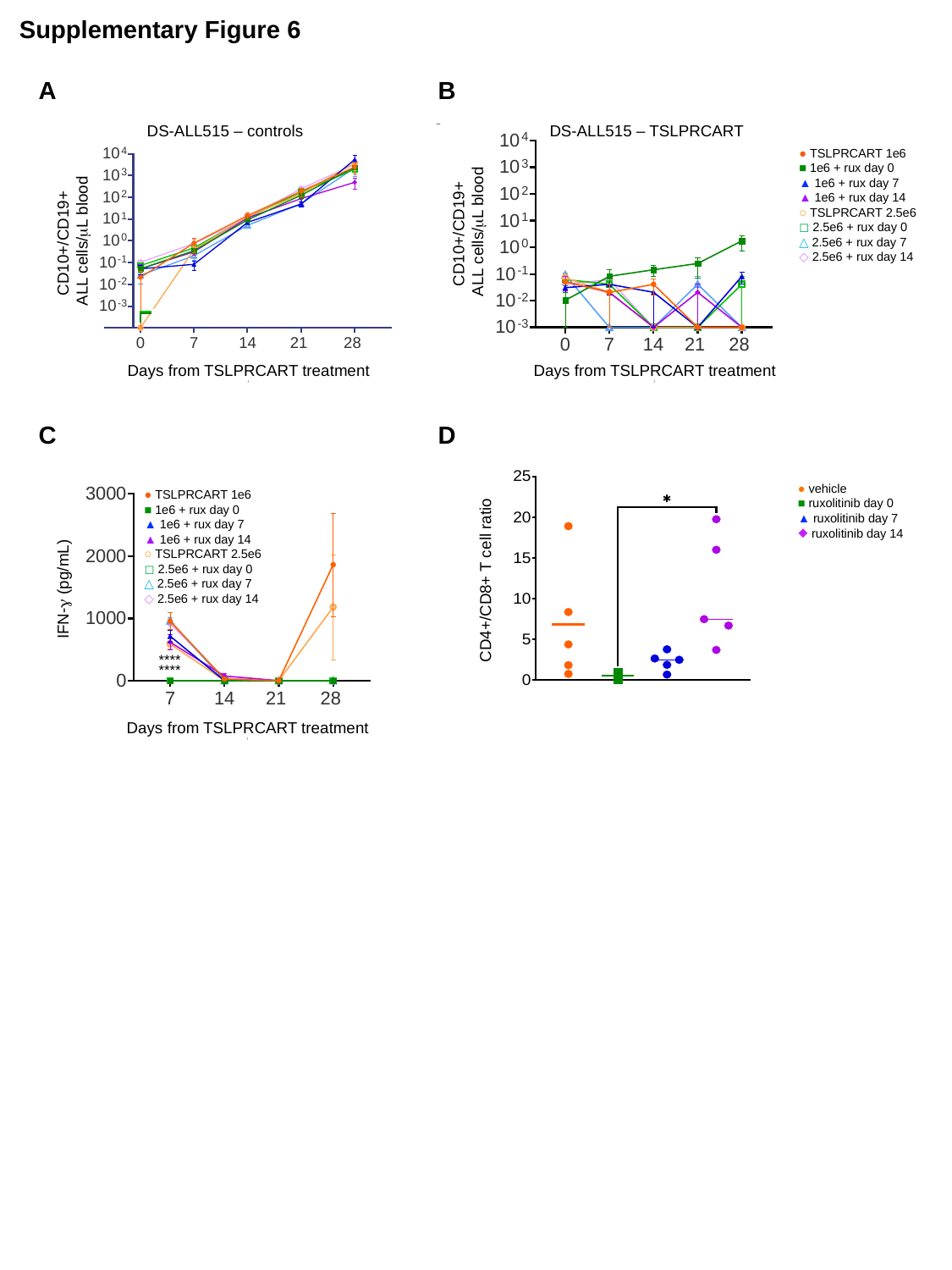

Supplementary Figure 6
A
B
DS-ALL515 – controls
DS-ALL515 – TSLPRCART
● TSLPRCART 1e6
■︎ 1e6 + rux day 0
▲ 1e6 + rux day 7
▲ 1e6 + rux day 14
○ TSLPRCART 2.5e6
◻︎ 2.5e6 + rux day 0
△ 2.5e6 + rux day 7
◇ 2.5e6 + rux day 14
CD10+/CD19+
ALL cells/mL blood
CD10+/CD19+
ALL cells/mL blood
Days from TSLPRCART treatment
/
Days from TSLPRCART treatment
/
C
D
● vehicle
■ ruxolitinib day 0
▲ ruxolitinib day 7
⬥ ruxolitinib day 14
CD4+/CD8+ T cell ratio
IFN-g (pg/mL)
Days from TSLPRCART treatment
/
● TSLPRCART 1e6
■︎ 1e6 + rux day 0
▲ 1e6 + rux day 7
▲ 1e6 + rux day 14
○ TSLPRCART 2.5e6
◻︎ 2.5e6 + rux day 0
△ 2.5e6 + rux day 7
◇ 2.5e6 + rux day 14
****
****

## Slide 7
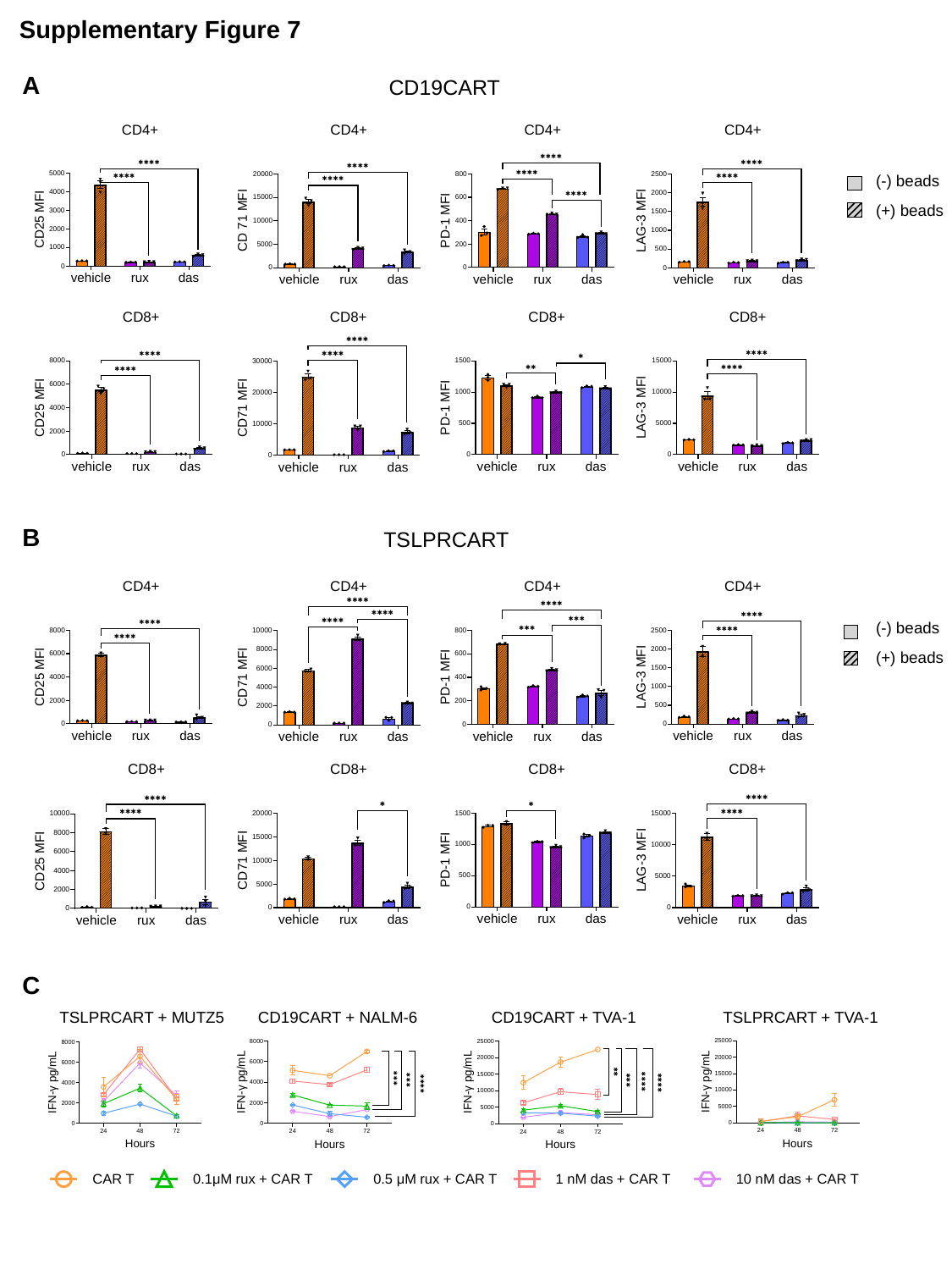

Supplementary Figure 7
A
CD19CART
(-) beads
(+) beads
B
TSLPRCART
(-) beads
(+) beads
C
TSLPRCART + MUTZ5
CD19CART + NALM-6
CD19CART + TVA-1
TSLPRCART + TVA-1
